# The *CaSBP11* gene functions as a negative regulator in pepper drought stress

**DOI:** 10.3389/fpls.2025.1497425

**Published:** 2025-04-28

**Authors:** Huai-Xia Zhang, Yuan Zhang, Bo-Wen Zhang, Fei-Fei Pan

**Affiliations:** College of Horticulture and Landscape Architecture, Henan Institute of Science and Technology, Xinxiang, Henan, China

**Keywords:** pepper, CaSBP11, drought stress, stomatal, ABA signaling pathway

## Abstract

The SBP-box gene family, an exclusively plant transcription factor, is critical for plant growth, development, and adaptive responses to both biotic and abiotic stresses. However, its role under non-biological stresses, specifically drought, remains overlooked in pepper plants. In our previous work, we isolated an SBP-box gene, *CaSBP11*, from the pepper genomic database. Subsequently, we employed virus-induced gene silencing, overexpression, and protein interaction experiments to investigate the function of *CaSBP11* under drought stress. Our results revealed that drought conditions significantly upregulated *CaSBP11* expression, whereas ABA treatment suppressed it. Silencing *CaSBP11* enhanced drought resistance in pepper, with increased stomatal aperture and ABA levels, and reduced stomatal density, water loss rates, and reactive oxygen species (ROS) accumulation compared to control plants. Conversely, overexpression of *CaSBP11* in *Nicotiana benthamiana* decreased drought tolerance, with *CaSBP11*-overexpressing plants showing reduced ABA sensitivity, lower stomatal aperture and ABA levels, and increased stomatal density and ROS production compared to wild-type plants. Interestingly, under non-stress conditions, core ABA signaling genes (*CaPP2C*, *CaPYL9*, *CaSNRK2.4*, *CaAREB*) exhibited lower expression in *CaSBP11*-silenced plants compared to controls, whereas this trend was reversed in *CaSBP11*-overexpressing lines (*NbPP2C*, *NbAREB*, *NbSNRK2.4*, *NbSRK2E*). Additionally, *CaSBP11* was found to interact with *CaPP2C*, *CaPYL9*, *CaSNRK2.4*, and *CaAREB* in nucleus. These data suggest that *CaSBP11* negatively regulates plant responses to drought stress and may interact with these key genes in the ABA signaling pathway to mediate this response.

## Introduction

Pepper, an important solanaceous vegetable, is highly nutritious and financially rewarding. However, it is susceptible to variousbiological and environmental stresses, including *Phytophthora capsici* infection, salinity, cold, and drought, all of which adversely affect crop yield (Feng et al., 2019). Drought, in particular, significantly impacts both the production and quality of pepper (Mahmood et al., 2021). To combat these biotic and abiotic stresses, plants activate intricate physiological and biochemical responses for adaptation (Hura et al., 2022; Zhang et al., 2023). Notably, stress responsive genes are triggered under most of such defense mechanisms and most importantly, regulated by transcription factors (Hirayama and Shinozaki, 2010). Numerous transcription factors in pepper have been identified as playing roles in the plant’s response to drought stress. For instance, the MYB transcription factor *CaDIM1* positively regulates the response to drought stress by modulating ABA-mediated gene expression (Lim et al., 2022). Additionally, the bZIP transcription factor CaATBZ1, a target protein of CaASRF1 whose stability is regulated by *CaASRF1*, negatively regulates the ABA signaling pathway and the pepper’s response to drought stress (Joo et al., 2019). Furthermore, CaATBZ1 can be ubiquitinated by CaATIR1 and interacts with it. Silencing of *CaATBZ1* can alleviate the sensitivity of CaATIR1-silenced plants to ABA and drought stress (Joo et al., 2020). Moreover, *CaDILZ1*, another member of the bZIP transcription factor family, shows increased expression levels following ABA treatment and interacts with *CaDSR1* to positively regulate the pepper’s response to drought stress (Lim et al., 2018a). The pepper dehydration-responsive homeobox domain transcription factor CaDRHB1 interacts with CaDSIZ1 and positively regulates plant responses to drought stress by modulating ABA-mediated stomatal closure (Lim et al., 2018b; Joo et al., 2022). The squamosa promoter Binding protein (SBP-box), a unique transcription factor in plants first discovered in *Antirrhinum majus*, containsa conserved SBP domain composed ofapproximately 76 amino acid residues, incorporating two canonical zinc finger proteins (Klein et al., 1996). Within the SBP-box gene family, some SBP genes harbor highly conserved miR156 recognition sites (Li et al., 2020). The SBP-box gene family is crucial for plant growth, morphogenesis, metabolic regulation, and stress adaptation, including hormone response, shoot branching, flower and fruit development, as well as biotic stress. For instance, during photoperiod-induced growth cessation in *Populus*, the photoperiodic perception mediated by the *FyB-PIF8* module correlates with the *miR156-SPL16*/*23*-*FT2*/*BRC1* regulatory cascade (Wei et al., 2024). *FHY3* and *FAR1* integrate photonic signals into the miR156-SPL module-modulated aging pathway, impacting *Arabidopsis* floral transition (Xie et al., 2020). In addition, *Arabidopsis thaliana AtSPL14* exhibits sensitivity to fumonisin B1 (Stone et al., 2005). Furthermore, *VpSBP5* is likely to be involved in regulating resistance to *Erwinia necator* in grape by inducing salicylic acid (SA) and methyl jasmonate (MeJA) molecular signals. The level of disease resistance in grapevine genotypes may correlate with the timing of the peak appearance of these signals (Hou et al., 2013). Additionally, the phosphorylated *IPA1/OsSPL14* triggers the expression of *WRKY45*, which in turn enhances resistance to rice blast (Wang et al., 2018). Besides, the SBP-box gene is also pivotal in abiotic stress responses. For instance, overexpression of *SPL6/7/9* genes in Sugarbeet confers enhanced drought resistance in transgenic plants (Wang et al., 2024). Overexpresion of *MiSPL3a*/*b* in *Arabidopsis* enhanced drought and ABA tolerance, yet Pro-Ca responsiveness remained high (Zhu et al., 2024). Silencing the *MsSPL9* gene in *alfalfa* enhances drought resistance by potentially regulating anthocyanin biosynthesis (Hanly et al., 2020). Besides, moderate miR156 expression attenuates *SPL13* gene expression, increasing *WD40–1* expression for drought resistance, while elevated miR156 expression impairs *alfalfa’s* drought resistance (Arshad et al., 2017; Feyissa et al., 2019). Moreover, in rice, knocking out the *OsSPL10* gene enhances the plant’s tolerance to salt stress (Lan et al., 2019). Overexpression of *VpSBP16* in *Arabidopsis* enhances the plant’s tolerance to both salt and drought stress (Hou et al., 2018). Furthermore, in *Arabidopsis*, *SPL9* regulates the plant’s response to freezing stress by modulating the expression of *CBF2* (Zhao et al., 2022). In wheat, silencing the *TaSPL6* gene enhancesdrought stress tolerance, while overexpressing *TaSPL6* reduces drought stress tolerance (Zhao et al., 2024). Additionally, the overexpression of *AhSPL5* in transgenic *Arabidopsis* can enhance salt tolerance by boosting its ROS-scavenging capability and positively regulating the expression of stress-responsive genes (Sun et al., 2024).There are 15 SBP-box genes in pepper, among which *CaSBP08*, *CaSBP11*, and *CaSBP12* negatively regulate the defense response of pepper to *Phytophthora capsici* infection (Zhang et al., 2016, 2018, 2020a, 2020b). Additionally, *CaSBP12* negatively regulates the defense response of pepper to salt stress (Zhang et al., 2020c). Furthermore, *CaSBP13* negatively regulates the defense response of pepper to drought stress (Zhang et al., 2024).

However, it appears that, beyond our research, the role of pepper SBP-box genes in drought stress is unreported (Zhang et al., 2024). Therefore, we explored the functionality of *CaSBP11* (Accession No. Capana10g000709) in pepper’s drought tolerance, building on our previous research (Zhang et al., 2016). The findings indicate that *CaSBP11* negatively regulates pepper’s response to drought stress.

## Materials and methods

### Plant materials and growing environments

The seeds for pepper cultivar AA3 and *Nicotiana benthamiana*(*N. benthamiana*)were obtained from the School of Horticulture Landscape Architecture at Henan Institute of Science and Technology, Xinxiang 453003, China. The pepper plants were grown in a controlled environment with a photoperiod of 16 hours of light and 8 hours of darkness, at temperatures of 22°C during dayand 18°C at night, and maintained at an optimal humidity level of 80%. Similarly, *N. benthamiana* was maintained at 25°C during the day and 18°C at night, with a preferred relative humidity of 60%.

### Silencing of *CaSBP11* in pepper

In compliance with the tobacco rattle virus-induced gene silence (VIGS) technology elucidated by Wang in 2013, the *CaSBP11* gene of pepper was silenced (Wang, 2013). The development of a virus-induced gene silencing test vector was based on the methodology by Zhang et al. (2020b). Specifically, a 224bp-specific fragment from the *CaSBP11* CDS region was amplified by specific primers (**Supplementary Table 1**), followed by cloning into TRV2 vector via a double enzyme digestion technique. The recombinant vector was sequenced by Shanghai Sangon Biotechnology Co., Ltd. and transferred to *Agrobacterium* strain GV3101 for storage.

During the formation of two true leaves in pepper seedlings, the silencing of the *CaSBP11* gene was performed using Zhang’s methodology with a prepared bacterial suspension containing the *CaSBP11* gene, and cultivated at 28°C until 0D600 = 0.8 (Zhang et al., 2013). It was mixed with an equal amount of TRV1 and inserted into the pepper cotyledon using a 1ml syringe without needles. After injection, the plants were maintained at 28°Cin darkness for 2 days.They were then cultured with a photoperiod of 16/8 hours, at temperatures of 22°C during the day and 18°C at night. Upon observing photo-bleaching in positive plants (TRV2:*CaPDS*), five random leaves from *CaSBP11-*silenced plants and control plants (TRV2:*00*) were chosen to assess the efficiency of silencing.

### Overexpression of *CaSBP11* in *N. benthamiana*

The vector used for *CaSBP11 N. benthamiana* overexpression was constructed based on the protocol outlined by Zhang et al. (2020b). Then, the re-engineered vector (pVBG2307:CaSBP11:GFP) was then employed for *CaSBP11* overexpression in *N. benthamiana*. *CaSBP11* overexpressing transgenic *N. benthamiana* were created through *Agrobacterium tumefaciens-*assisted leaf disc transformation (Oh et al., 2005). RNA analysis confirmed two kanamycin-resistant *CaSBP11* transformants. The T1 progeny originated from T0 plant regeneration, with T2 progeny deriving from T1 plants. T3 progeny were chosen for subsequent research.

### Stress treatments and samples collection

To evaluate the expression of the *CaSBP11* gene in peppers under drought stress, 6–8 true leaf seedlings were obtained from substrate (constructed with a 3:1:1 blend of matrix, perlite, and vermiculite).The seedlings were then cultivated in 1/2 Hoagland’s solution, with 20% Polyethylene glycol (PEG6000) applied for three days. Control cultures remained solely in 1/2 Hoagland’s solution. Leaves were collected at 0 h, 3 h, 6 h, 12 h, and 24 h, and stored at −80°C.

For drought treatments of CaSBP11-silenced plants and *CaSBP11* overexpression plants, procedures outlined by Zhang et al. (2024) are applied. CaSBP11-silenced and control plants, as well as *CaSBP11* overexpression and wild-type plants, were cultivated under identical controlled conditions prior to and during drought treatment. The conditions for CaSBP11-silenced and control plants were 16 hours of light, 8 hours of darkness, 22°C during the day, and 18°C at night, with a humidity of 80%. For *CaSBP11* overexpression and wild-type plants, the conditions were 16 hours of light, 8 hours of darkness, 25°C during the day, and 18°C at night, with a humidity of 60%. Prior to drought treatment of CaSBP11-silenced and control plants, sufficient irrigation is implemented one month ahead with watering repeated every third day until the final watering, marking the onset of drought stress (Day 0) three days past the treatment commencement. Sampling is performed on days 0, 1, 2, 3, and 4, with samples are stored at −80°C for future use. For drought treatment in *CaSBP11* overexpressed and wild-type plants, the identical procedures involve pre-treating with sufficient irrigation, followed by watering every four days until the final watering, marking the onset of drought stress (day 0). Sampling is conducted on days 0, 2, and 4, with samples being stored at −80°C for future use.

For ABA treatment, seedlings were exposed to 20μM ABA in accordance with Yin et al. (2014). Controls consisted of a 0.5% tween and 0.1% ethanol solution. Leaves were harvested at 0h, 3h, 6h, 12h, 24h, and 48h, and stored at −80°C for future use. The statistical data for germination and root length of *CaSBP11* overexpressed plants are performed according to the procedure delineated by Ma et al. (2011). Four ABA concentration gradients (0g/L ABA, 0.1g/L ABA, 0.5g/L ABA, and 1.0g/L ABA) were used for these experiments. The seeds of *CaSBP11*-overexpression plants were soaked in these ABA solutions for 24 hours, then placed on a culture dish with two layers of moistened filter paper under 25°C, 16 hours light, and 8 hours dark conditions. Ventilation was performed daily for 10 minutes, and water was added to maintain moisture. During the third, fifth, and tenth days, germination data were documented. For the root length statistics of *CaSBP11*-overexpression plants, the same method was employed, with the root length being counted on the tenth day.

### RNA extraction and quantitative real-time PCR

Total RNA was extracted via the RNAprep Pure Micro Kit (Tiangen Beijing, China), in accordance with manufacturer’s guidelines. Reverse transcription was accomplished with the transcriptor First Strand cDNA Synthesis Kit (Tiangen Beijing, China). Diluted cDNA was used for quantitative real-time (qPCR) analysis at a concentration of 50 ng/L. Utilizing the iQ5.0 Bio-Rad iCycler thermocycler (Bio-Rad, Hercules, CA, USA), we implemented real-time Qpcr according to the methodology outlined by Zhang et al. (2020a). This involved a pre-denaturation phase at 95 °C for 1 min, followed by 40 cycles of denaturation (95 °C, 10 s), annealing (56 °C, 30 s), and extension (72°C, 30s). End-of-cycle fluorescence detection ensured PCR primer accuracy through post-PCR profile analysis ranging from 56 to 95 °C. The specificity of all primers was rigorously validated using NCBI Primer BLAST (**Supplementary Table 1**). Gene expression was assessed and standardized against *CaUBI3* (GenBank: AY486137.1), and *Nbactin-97* (GenBank: XM_019369243.1) (Schmittgen and Livak, 2008; Du et al., 2015; Zhang et al., 2016).

### Drought index percentage analysis

Percentage drought index analysis employed Zhang’s methodology (Zhang et al., 2020b). Upon 5 days of drought stress, the drought phenotypes of CaSBP11-silenced and control plants were classified, and the percentage drought indices of both groups were calculated. The classification criteria were as follows: level 0, no symptoms; level 1, lower leaves wither; level 2, all leaves except the growing point wither; level 3, entire plant withers. For drought stress of 13 days, the drought phenotypes of *CaSBP11* overexpression and wild-type plants were classified, and the percentage drought indices of both groups were calculated. The classification criteria were as follows: level 0, no symptoms; level 1, lower leaves wither or yellow; level 2, lower leaf death; level 3, entire plant death except the growing point.

### Determination of physiological indicators

Malondialdehyde (MDA), relative water content, and relative electrical conductivity evaluations followed Zhang et al. (2018) and Pan et al. (2012) protocols. Water loss rate was calculated based on Ma et al. (2021) method. Total chlorophyll content was determined via Arkus et al. (2005) methodology. Assays for peroxidase (POD), catalase (CAT) and superoxide dismutase (SOD) activities were conducted following Zhang et al. (2018); Stewart and Bewley (1980) methodology.

For hydrogen peroxide (H_2_O_2_) and oxygen (O_2_^-^) radical analysis, diaminobenzidine (DAB) and nitrotetrazolium blue chloride (NBT) stains were utilized as per Choi and Hwang (2012) and Kim et al. (2012) protocol, respectively. Quantification of these stained regions was executed based on Sekulska-nalewajko et al. (2016),while H_2_O_2_ content determination utilized Liu et al. (2010). Superoxide anion (O_2_^-^) detection is accomplished in accordance with the Solarbio Superoxide Anion kit guidelines (Solarbio, Beijing, China).

The analysis of ABA content utilizes the Plant Hormone Abscisic Acid (ABA) Enzyme Immuno Fluorometric Assay Kit (KeLu, Wuhan, China). The procedure is carried out as per the enclosed instructions.

The stomatal morphologies were observed at high magnification using a scanning electron microscope (FEI Quanta 200, USA). The stomatal density was accurately measured via the NIH (National Institutes of Health)-endorsed Image J software Stomatal apertures were determined via SEM, with the aperture dimensions encompassing both pore length (dumbbell-shaped aperturae) and width (maximum perpendicular to the dumbbell-shaped aperturae). Besides, SEM examination unveiled the dimensions of the stomatal apertures. The stomatal aperture encompasses both the pore length and width of the pore. The dumbbell-shaped aperturaeis identified as the latter, while its maximal perpendicular value represents the former.

### Bimolecular fluorescence complementation and co-immunoprecipitation experiments

The vectors of pSPYCE-35S and pSPYNE-35S were used for these experiments. Besides, the target gene was ligated into the vector using the homologous recombination method, and the primers for vector construction are listed in **Supplementary Table 1**. The constructed recombinant vectors were transferred into *Agrobacterium* (GV3101) for the BiFC and Co-IP experiments. For the BiFC experiment, the above transformed *Agrobacterium* bacterial solution was cultured overnight at 28°C and 200 rpm until the optical density (OD) reached 0.5. The culture was then centrifuged, and the supernatant was discarded. The pellet was re-suspended in an equal volume of suspension buffer. After resuspension, the bacterial solution was left to stand at room temperature for 3 hours. The bacterial suspension was then injected into *Nicotiana Benthamiana* leaves using a 1 ml syringe without a needle. After injection, the plants were maintained in darkness at 28°C for one day, followed by cultivation under a photoperiod of 16/8 hours, with temperatures of 22°C during the day and 18°C at night. Fluorescence was observed using a confocal laser scanning microscope (FV10-ASW, Olympus Corporation, Japan) three days after infection. The excitation wavelengths used were 515 nm for the yellow fluorescence field, 488 nm for the chloroplast auto fluorescence field, and 358 nm for the DAPI field (nuclear staining). The Co-IP experiments were conducted according to the method described by Song et al. (2023).

### Statistical analysis

The SPSS 22.0 statistical software was utilized to evaluate the effects of different treatment alterations using a one-way ANOVA test. *Post hoc* Tukey analysis revealed potential significant differences at *P ≤* 0.05 and *P ≤* 0.01. Data is presented as mean ± standard deviation (SD). Experiments necessitate at least three biological replicas in rigorously designed protocols.

## Results

### Expression of the *CaSBP11* gene during drought and ABA stress in pepper

To investigate the role of *CaSBP11* in resilience to drought and ABA stress its expression pattern in pepper was analyzed. As shown in **Supplementary Figure 1**, *CaSBP11* transcripts initially decreased at 3h post drought stress, subsequently significantly increasing at 12h. Besides, the expression of *CaSBP11* was suppressed during ABA treatment (**Supplementary Figure 1**). These findings indicating that *CaSBP11* responds to both drought and ABA stress.

### *CaSBP11* gene silencing enhances pepper plant drought resilience

Virus-induced gene silencing was applied for elucidation of *CaSBP11*’s function in pepper’s drought stress response (Wang, 2013).The established positive control,TRV2:*CaPDS*, silenced the *CaPDS* gene, resulting in photo-bleached leaf symptoms, while TRV2:*00* served as the negative control. Upon observing photo-bleaching in positive control plants, the silencing efficiency of TRV2:*CaSBP11* was evaluated (**Supplementary Figure 2**). The results in **Supplementary Figure 2** show that CaSBP11-silenced (TRV2:*CaSBP11*) and control (TRV2:*00*) plants displayed no remarkable phenotypic variations under regular conditions. Additionally, the silencing efficiency of the *CaSBP11* gene exceeds 84%. Therefore, both CaSBP11-silenced and control plants were used for subsequent analyses.

**Figure 1:**
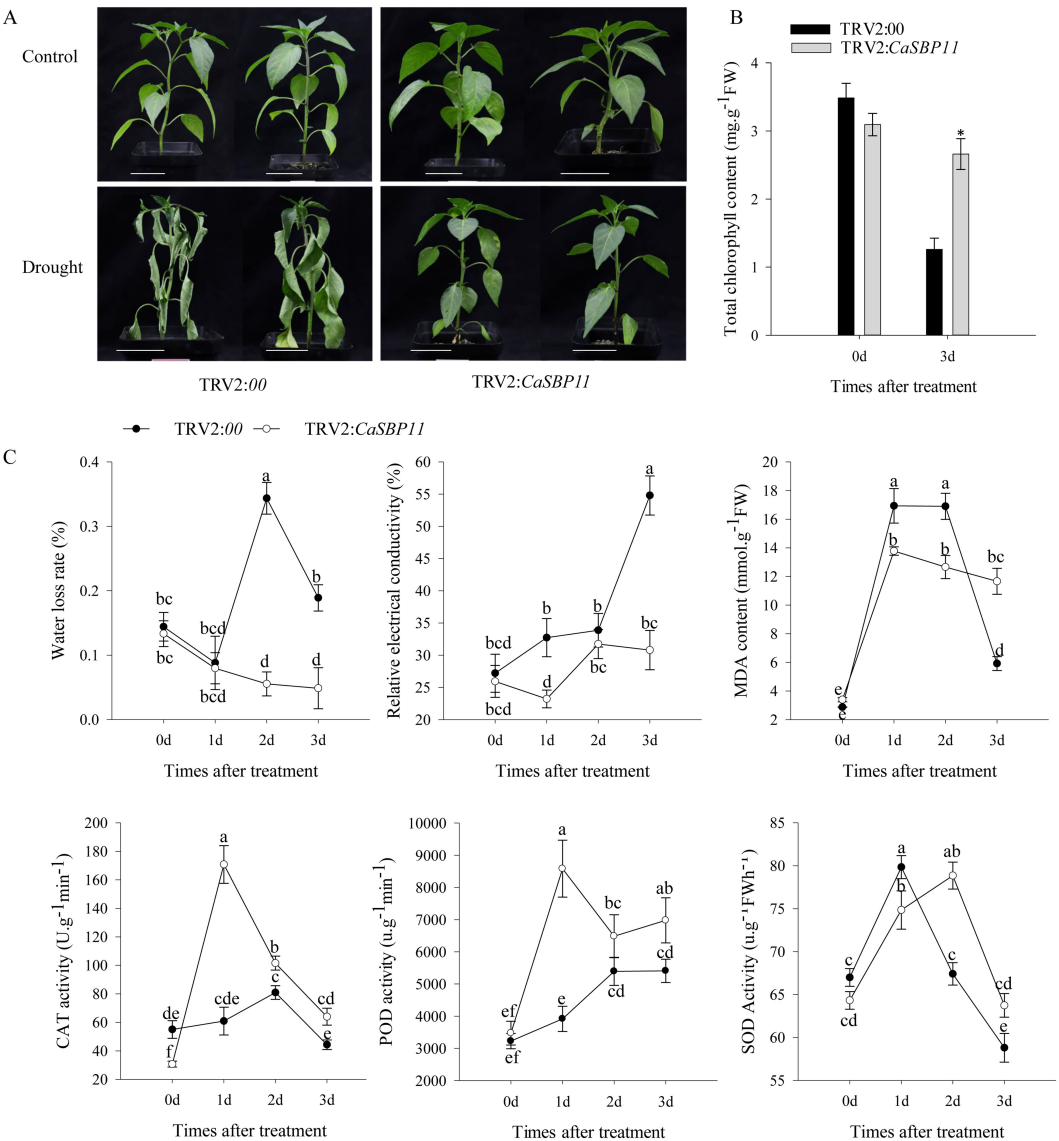
Investigating drought resilience in CaSBP11-silenced plants. **(A)** Drought-induced morphological changes of CaSBP11-silenced and control plants at three days post-drought stress, alongside their respective non-stressed conditions. Scale bar, 3.5 cm. **(B)** Chlorophyll content of CaSBP11-silenced versus control plants under drought stress. **(C)** Post-drought stress, the water loss rate, relative electrical conductivity, MDA concentration, SOD, CAT, and POD activities of CaSBP11-silenced and control plants d: day. * Denotes significance at *P* ≤ 0.05. Letters denote significant differences at *P* ≤ 0.05. Mean values and SDs for three replicates are displayed.

**Figure 2:**
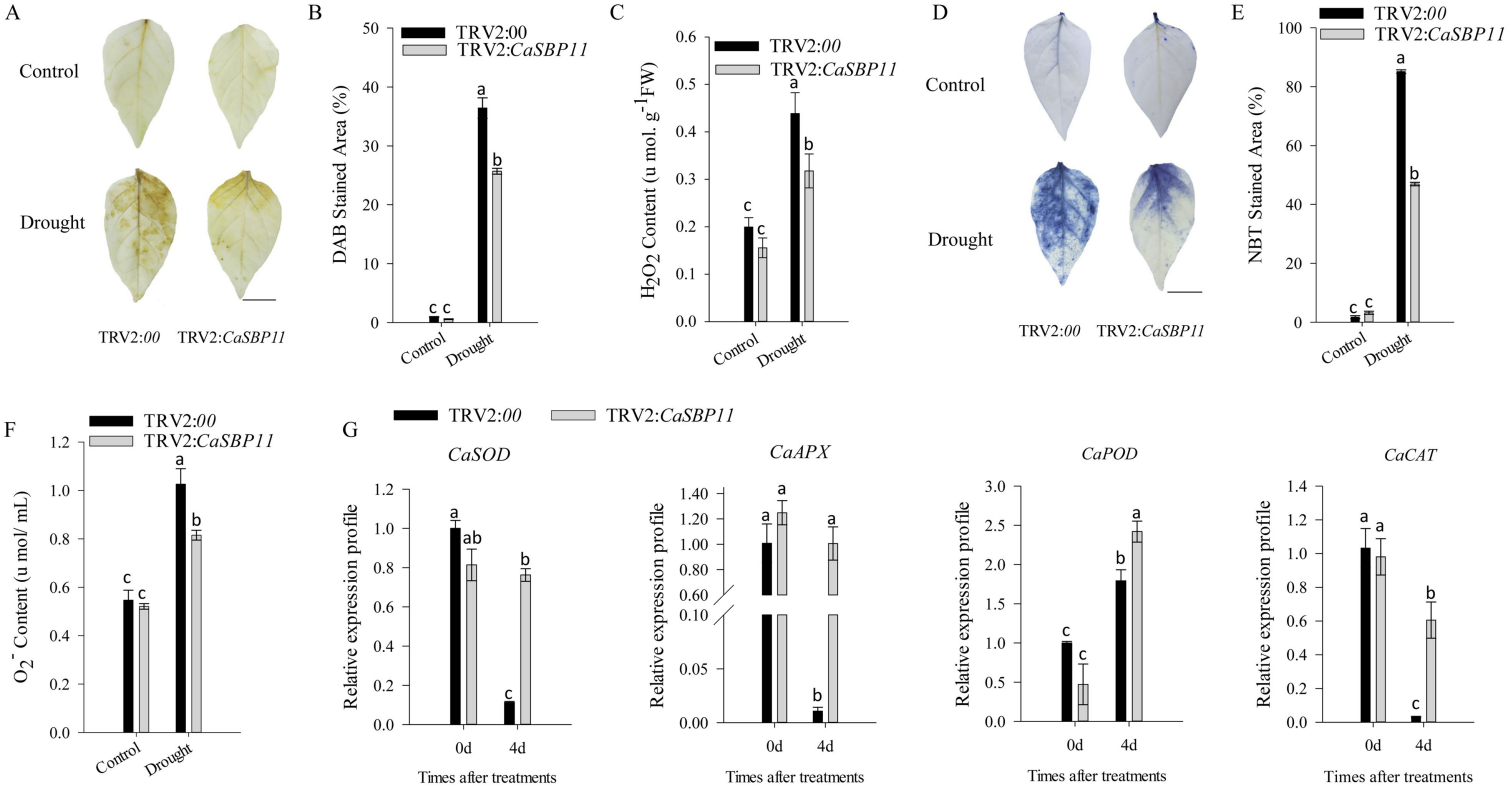
Characterization of CaSBP11-silenced versus control plants through DAB and NBT staining, evaluation of hydrogen peroxide and superoxide anion levels, and monitoring of ROS-scavenging enzyme gene expression. **(A)** DAB staining in drought-stressed leaf tissue after four days. Scale bar, 0.6 cm. **(B)** DAB-stained region in leaves following drought stress. **(C)** Post-drought stress hydrogen peroxide content in CaSBP11-silenced versus control plants.**(D)** NBT staining in drought-stressed leaf tissue after four days. Scale bar, 0.6 cm. **(E)** NBT-stained region in leaves post-drought stress. **(F)** Post-drought stress superoxide anion content in CaSBP11-silenced versus control plants. **(G)** Expression of ROS-scavenging enzyme genes post-drought stress in CaSBP11-silenced and control plants. Different letters denote significant differences at P ≤ 0.05. Mean values and SDs for three replicates are displayed.

After enduring three drought days, CaSBP11-silenced plants demonstrated negligible phenotypic modifications while controls exhibited leaf wilting symptoms, including chlorosis in lower foliage (**Figure 1A**). Comparatively, there were no discernible differences in the phenotype of non-stressed CaSBP11-silenced and control plants (**Figure 1A**). Upon drought exposure, chlorophyll content decreased in both CaSBP11-silenced and control plants, yet the former retained significantly more (**Figure 1B**).Additionally, the rate of water loss in CaSBP11-silenced plants showed a decreasing trend after drought, notably outperforming controls at two and three days (**Figure 1C**). The electrical conductivity of CaSBP11-silenced plants varied consistently below the control plants, particularly on Days 1 and 3 (**Figure 1C**). Both plant types exhibited an overall increase in MDA content, with control plants showing higher values on Days 1 and 2 (**Figure 1C**). Besides, CaSBP11-silenced plant samples exhibited superabundant CAT and POD activities on Day 1, achieving statistical significance (**Figure 1C**). SOD activity exhibits a trend of initial increase and subsequent decline, while the peak time point in control plants precedes that of CaSBP11-silenced plants (**Figure 1C**). Upon drought stress for 5 days, we classified the drought phenotypes of CaSBP11-silenced and control plants to calculate their percent drought indices. The classifying criteria were: level 0, no symptoms; level 1, lower leaves of plant wilted; level 2, all plant leaves except the growing point wilted; level 3, entire plant wilted (**Supplementary Figure 3A**). Subsequently, we statistically analyzed the percent drought indices of CaSBP11-silenced and control plants. As demonstrated in **Supplementary Figure 3B**, at 5 days of drought stress, the drought index of CaSBP11-silenced plants was notably lower than that of control plants. These results indicated improved drought endurance in the CaSBP11-silenced plants. Subsequently, to assess reactive oxygen species (ROS) accumulation in drought-stressed plants with CaSBP11-silenced and control groups, H_2_O_2_ and O_2_^-^ were detected via DAB and NBT staining (**Figures 2A, D**). After four days of drought exposure, significant DAB and NBT staining area was observed in control leaves compared to CaSBP11-silenced leaves (**Figures 2B, E**), with elevated H_2_O_2_ and O_2_^-^content (**Figures 2C, F**). This evidence suggests reduced ROS accumulation in CaSBP11-silenced foliage relative to control leaves. Furthermore, gene expression levels pertinent to ROS removal (*CaAPX1*, *CaPOD*, *CaSOD*, and *CaCAT2*) were evaluated (**Figure 2G**). On the fourth day of drought stress, except for elevated *CaPOD* expression, gene expression in CaSBP11-silenced and control plants decreased (**Figure 2F**). Nonetheless, these genes displayed significantly higher expression in CaSBP11-silenced plants compared to controls (**Figure 2G**).

**Figure 3:**
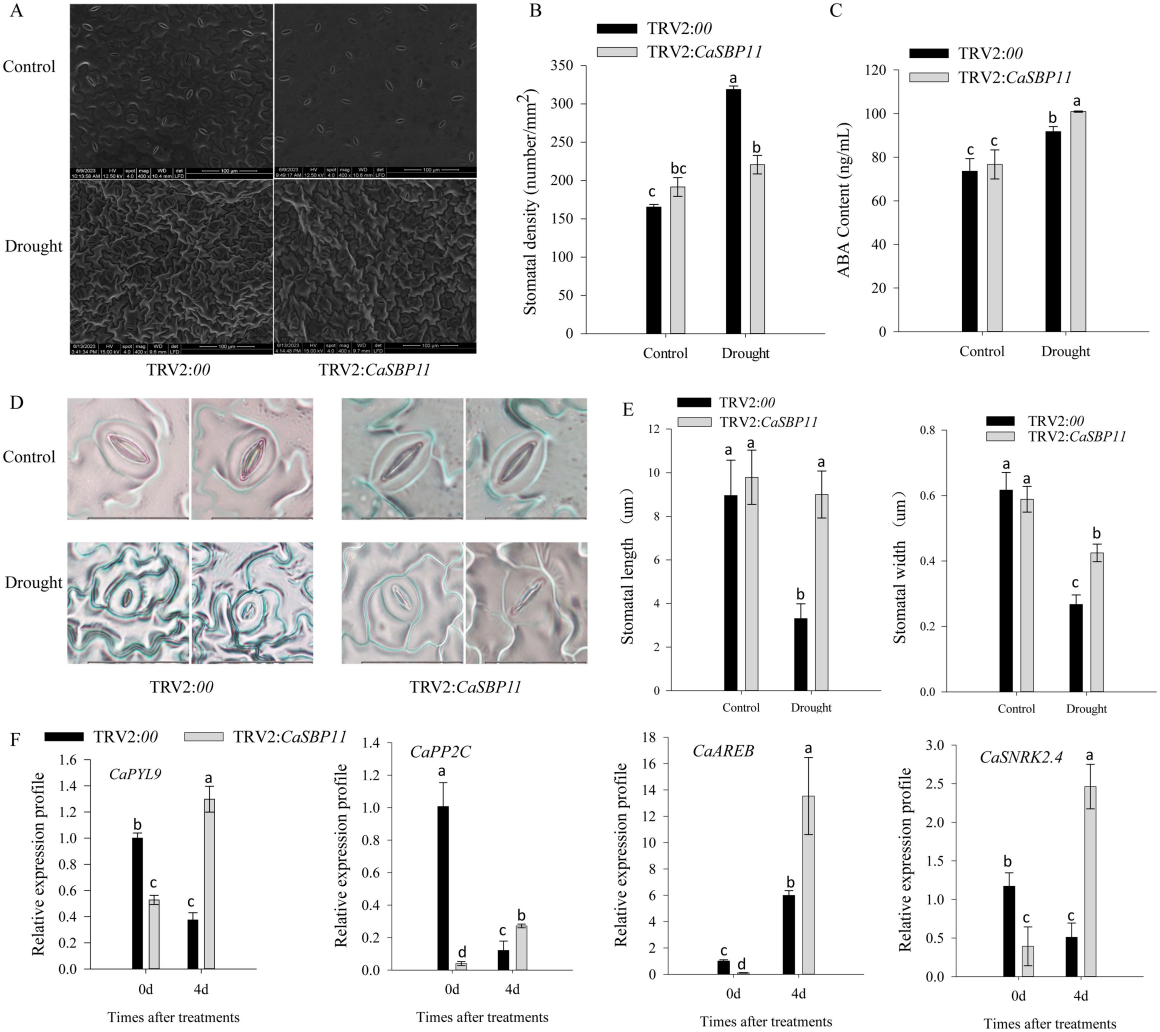
The stomatal state, ABA concentration, and key gene expression of the ABA signaling pathway in drought-stressed CaSBP11-silenced and control plants. **(A)** Stomatal morphometric data for both groups. **(B)** Estimation of stomatal density. Scale bar, 100 µm. **(C)** ABA content of both groups. **(D)** Stomatal morphologic traits. Scale bar, 20 µm. **(E)** Stomatal length/width. **(F)** Key gene expression of ABA signaling pathway post drought in both groups. Different letters denote significant differences at P ≤ 0.05. Mean values and SDs for three replicates are displayed.

The stomatal density of control plants was significantly higher than that of CaSBP11-silenced plants (**Figures 3A, B**). During four-day drought stress, ABA content increased in both CaSBP11-silenced and control plants; however, the increase was significantly greater in the former (**Figure 3C**). Additionally, post-drought stress, stomatal length and width decreased, but CaSBP11-silenced plants still exhibited noticeably larger dimensions compared to the controls (**Figures 3D, E**). Furthermore, key genes in the ABA signaling pathway (*CaSNRK2.4*, *CaPYL9*, *CaAREB*, and *CaPP2C*) were analyzed as shown in **Figure 3F**. These genes displayed elevated expression levels in CaSBP11-silenced plants under drought stress after four days (**Figure 3F**). Notably, even when untreated, their expression levels were lower in the CaSBP11-silenced plants than in the controls (**Figure 3F**).

### *CaSBP11* overexpression notably diminishes drought resistance in *N. benthamiana*

To better understand *CaSBP11*’s role in plant drought resilience, we generated two transgenic lines (line 9 and line10) overexpressing this gene in *N. benthamiana*. The gene expression level in these lines is illustrated in **Supplementary Figure 4**. In the absence of ABA, there was no discernible difference in germination rates between wild-type and *CaSBP11* overexpression seeds (**Supplementary Table 2**). However, under ABA treatment, the germination rates of *CaSBP11* overexpression seeds exceeded those of wild-type seeds (**Supplementary Table 2**). Specifically, at 0.1g/L ABA, *CaSBP11* overexpression seeds demonstrated a distinctly increased germination rate at day 3 when compared to wild-types. Similarly, at 0.5g/L ABA, *CaSBP11* overexpression seeds displayed a substantially superior germination rate when compared to wild-types by the third day. Lastly, under 1g/L ABA, *CaSBP11* overexpression seeds demonstrated markedly enhanced germination rates compared to wild-types by day 5 (**Supplementary Table 2**). Moreover, root length of *CaSBP11* overexpression plants and wild-types diminished as the treatment intensity elevated across different concentration gradients. However, exposure to 0.1g/L ABA, 0.5g/L ABA, and 1g/L ABA significantly enhanced the root length of *CaSBP11* overexpression plants compared to wild-types (**Supplementary Figures 5A, B**). These data show overexpression of *CaSBP11* in *N.benthamiana* diminishing plant sensitivity to ABA.

**Figure 4:**
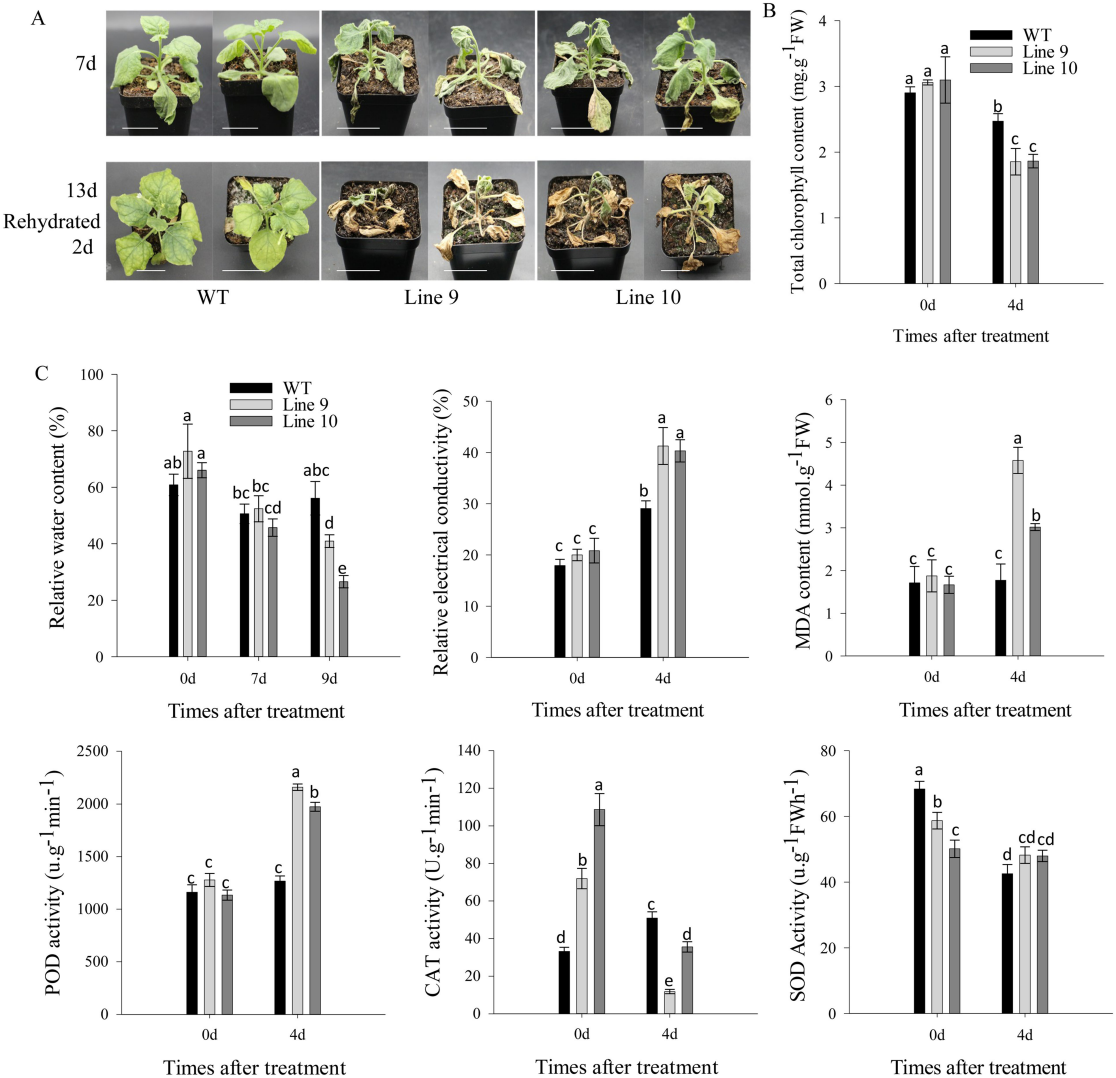
*CaSBP11* overexpression in *Nicotiana Benthamiana* heightens drought susceptibility. **(A)** Upper layer illustrates the phenotype of *CaSBP11* overexpressed and wild-type (WT) lines during seven days of drought, lower layer depicts their status after two days post rehydration following a thirteen days drought period. Scale bar, 3.5 cm. **(B)** Four days post-drought stress, *CaSBP11* overexpression and wild-type total chlorophyll content. **(C)** Four days post -drought stress, *CaSBP11* overexpression versus wild type relative water content, relative electrical conductivity, and malondialdehyde levels. Different letters denote significant differences at P ≤ 0.05. Mean values and SDs for three replicates are displayed.

Additionally, post-seven-day drought exposure, both *CaSBP11* overexpression and wild-type plants displayed wilt (**Figure 4A**). However, the transgenic plants exhibited pervasive wilt and severe yellowing of lower leaves, while the wild-type ones only experienced wilt of lower leaves (**Figure 4A**). After 13 days of drought, when rewatering occurred, nearly all *CaSBP11* overexpression plants, apart from the growing point, had died. In contrast wild-type plants displayed no growth anomalies except for leaf yellowing (**Figure 4A**). Besides, we categorized the drought phenotypes of *CaSBP11* overexpression plants and wild-type plants after 13 days of drought stress. The classification criteria were as follows: level 0, no symptoms; level 1, wilting or yellowing of lower leaves; level 2, sublethal leaf death; level 3, plant death excluding the growing point (**Supplementary Figure 6A**). Subsequently, we calculated the percentage of drought index of *CaSBP11* overexpression plants and wild-type plants. As shown in **Supplementary Figure 6B**, the percentage of drought index of *CaSBP11* overexpression plants was notably elevated compared to wild-types. Additionally, at day 4 of drought, *CaSBP11* overexpression plants displayed significantly reduced chlorophyll content compared to wild-types (**Figure 4B**). Similarly, their MDA content and relative electrical conductivity showed a significant increase (**Figure 4C**). Additionally, plants overexpressing *CaSBP11* displayed a noticeable decrease in relative water content on day 9 of drought stress exposure, as depicted in **Figure 4C**. These data underscore improved drought stress sensitivity in *CaSBP11* overexpressing plants.

**Figure 5:**
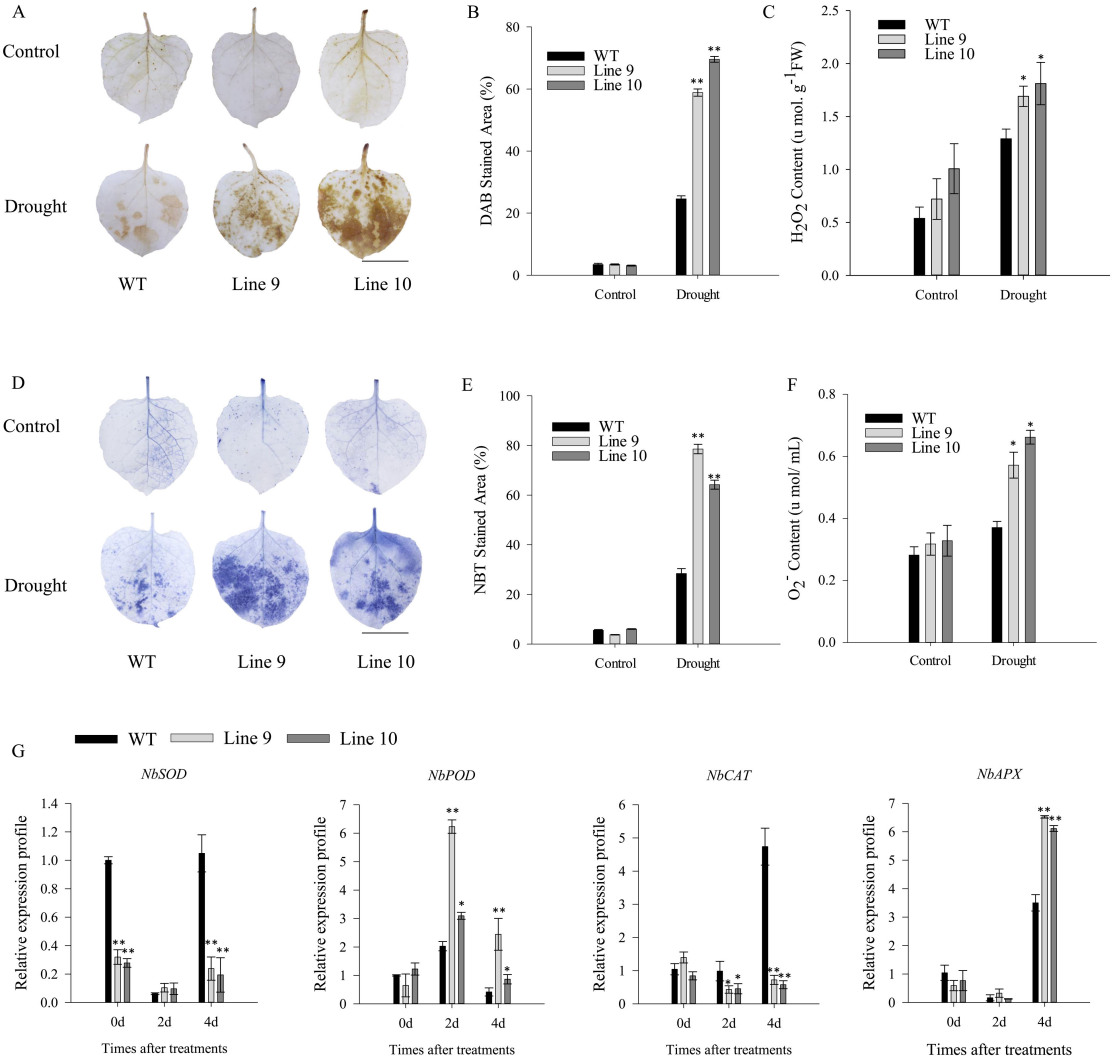
Assessing *CaSBP11* overexpressed and wild-type lines via DAB and NBT staining, coupled with analysis of hydrogen peroxide and superoxide anion contents, and expression of ROS-scavenging enzyme genes following drought stress. **(A)** DAB staining in leaf tissues exposed to four-day drought. Scale bar, 1.4 cm. **(B)** DAB stain area for these lines after drought. **(C)** H_2_O_2_ content for both lines post-drought stress. **(D)** NBT staining in leaf tissues post-drought stress. Scale bar, 1.4 cm. **(E)** NBT stain area for both lines post drought-stress. **(F)** O_2_^-^ content for both lines post-drought stress. **(G)** Expression of ROS-scavenging enzyme genes post-drought stress. * and ** denote significant differences at *P* ≤ 0.05 and *P* ≤0.01 respectively. Mean values and SDs for three replicates are displayed.

In addition, on day 4 of drought stress, the *CaSBP11* overexpression plants exhibited larger DAB and NBT staining regions than wild-types (**Figures 5A, B, D, E**). Their H_2_O_2_ and O_2_^-^content was appreciably higher than wild-type plants (**Figures 5C, F**). These data suggest a potential role for *CaSBP11* in ROS signaling pathways during drought stress. Therefore, gene expressions associated with the ROS pathway (*NbPOD*, *NbCAT*, *NbAPX*, *NbSOD*) were examined. During drought, *NbPOD* expression was induced and notably elevated compared to wild-type (**Figure 5G**). Conversely, *NbCAT* expression in the *CaSBP11* overexpression plants was notably reduced (**Figure 5G**). Moreover, during the fourth day of drought stress, *NbAPX* expression in *CaSBP11* overexpressing plants displayed notably higher levels compared to wild-types (**Figure 5G**). However, *NbSOD* expression in these overexpressing plants is notably reduced (**Figure 5G**)

**Figure 6:**
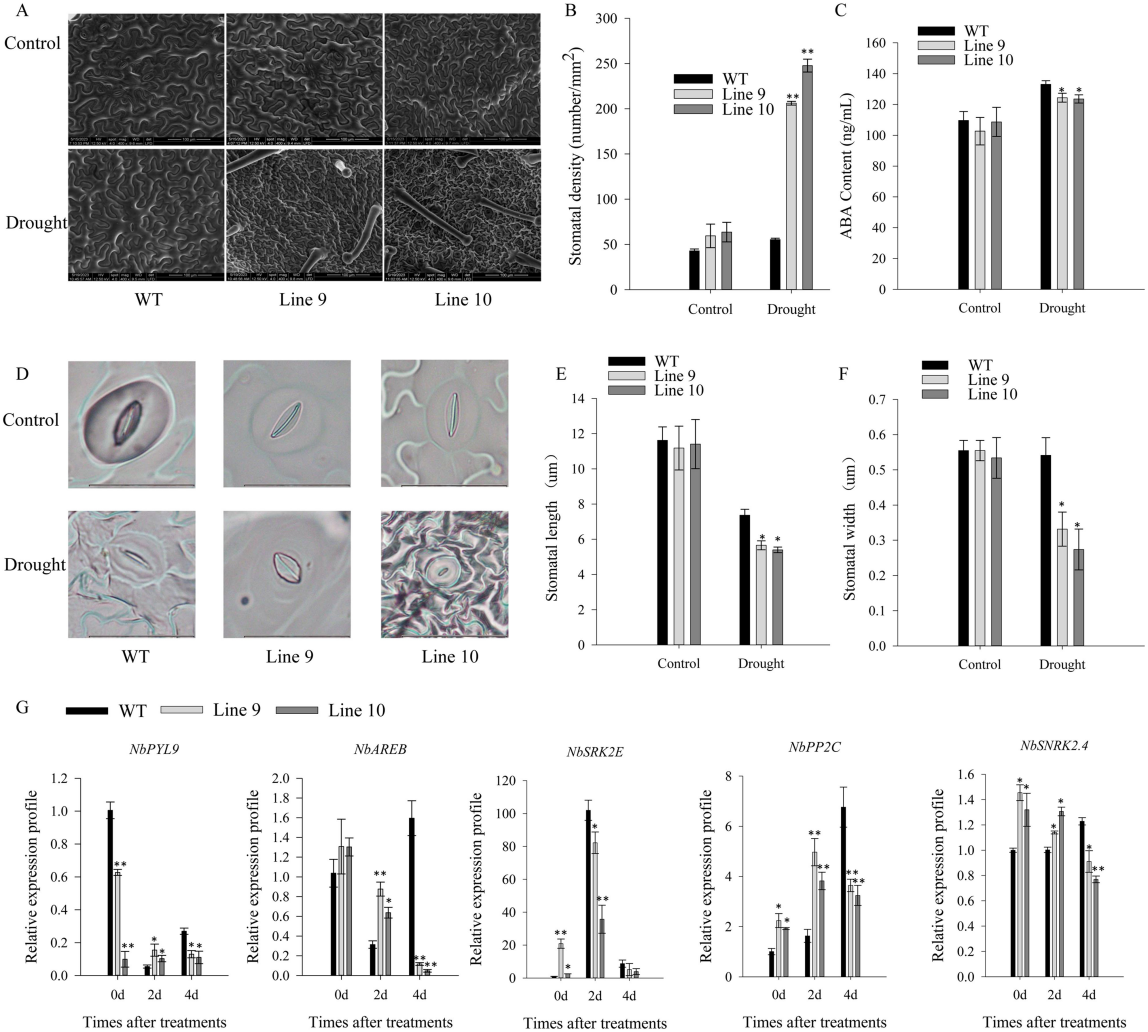
Stomatal state, ABA concentration, and key gene expression of the ABA signaling pathway in drought-stressed *CaSBP11* overexpressed and wild-type plants. **(A)** Stomatal morphologies of *CaSBP11* overexpressed plants and wild types. **(B)** Stomatal density evaluation. Scale bar, 100 µm. **(C)** ABA levels in *CaSBP11* overexpressed plants and wild types. **(D)** Stoma morphologies. Scale bar, 20 µm. **(E)**, **(F)** Stoma length/width. **(G)** ABA signaling pathway key genes’ expression of *CaSBP11* overexpressed plants and wild-types post-drought stress. * and ** denote significance at *P* ≤ 0.05 and *P ≤* 0.01 respectively. Mean values and SDs for three replicates are displayed.

Besides, the stomatal density of *CaSBP11* overexpression plants significantly exceeded that of their wild-type counterparts (**Figures 6A, B**). Similarly, the ABA concentration in *CaSBP11* overexpression plants significantly declined compared to wild-types (**Figure 6C**). Notably, drought stress resulted in diminished pore size in both *CaSBP11* overexpression and wild-type plants after four days, with a remarkable disparity in pore length and width between these strains (**Figures 6D–F**). During this time, significant reductions in the transcript levels of key ABA signaling pathway genes like *NbPYL9*, *NbAREB*, *NbPP2C*, and *NbSNRK2.4* were observed in the *CaSBP11* overexpression plants compared to wild-types (**Figure 6G**). Notably, without treatment, the *CaSBP11* overexpression plants exhibited higher expressions of *NbPP2C*, *NbSNRK2.4*, and *NbSRK2E* versus wild-types (**Figure 6G**). These results indicate that overexpression of *CaSBP11* in *N.benthamiana* accentuates plant drought stress susceptibility, potentially linked to ROS and ABA signaling pathways.

### CaSBP11 interacts with CaPP2C, CaPYL9, CaSNRK2.4, and CaAREB

**Figure 7:**
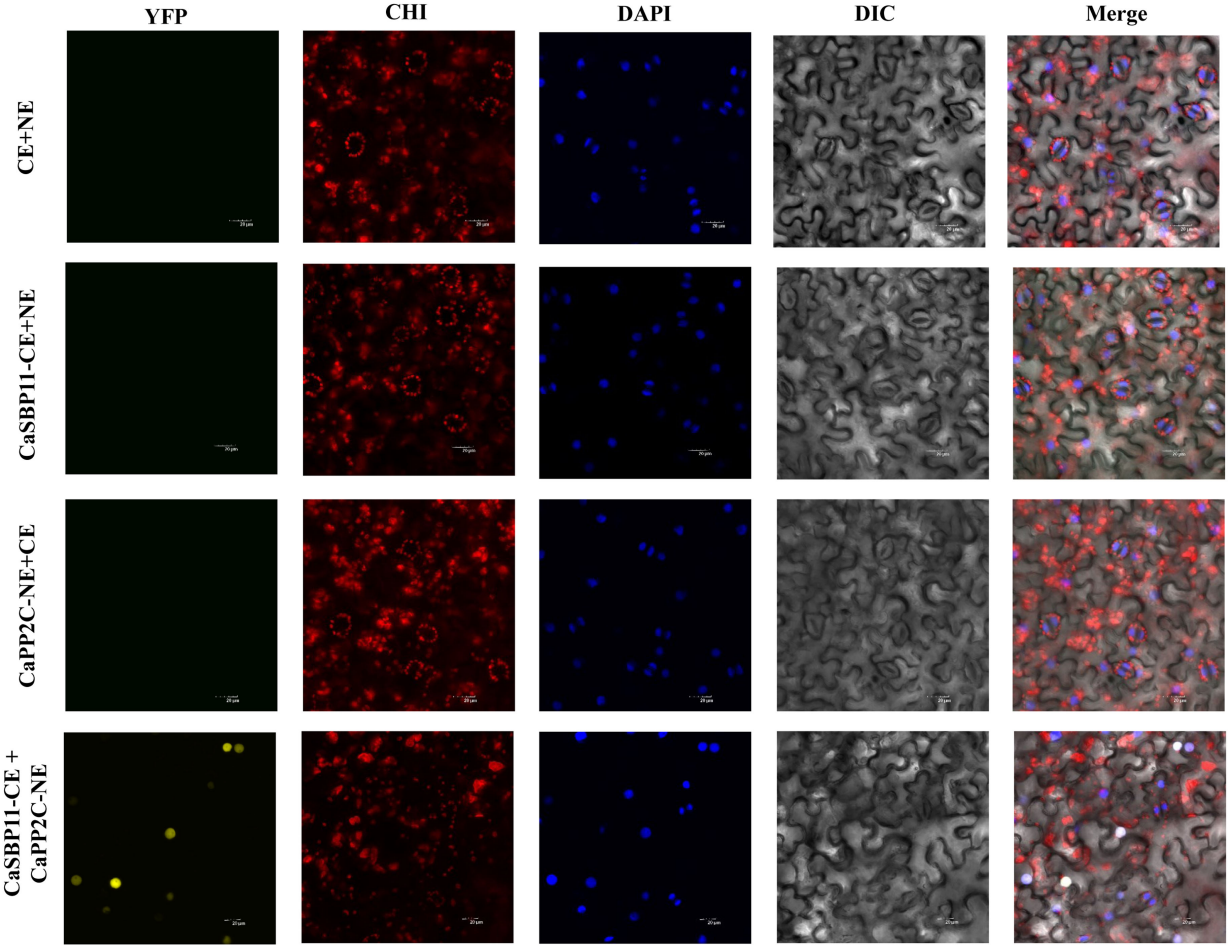
BiFC assay of CaSBP11 and CaPP2C. YFP represents the yellow fluorescent field, CHI represents the chloroplast autofluorescence field, DAPI represents the DAPI field (nuclear staining), DIC represents the bright field, and Merge represents the overlay field. Excitation wavelengths: YFP field (515 nm), CHI field (488 nm), DAPI field (358 nm). Bar = 20 µM.

**Figure 8:**
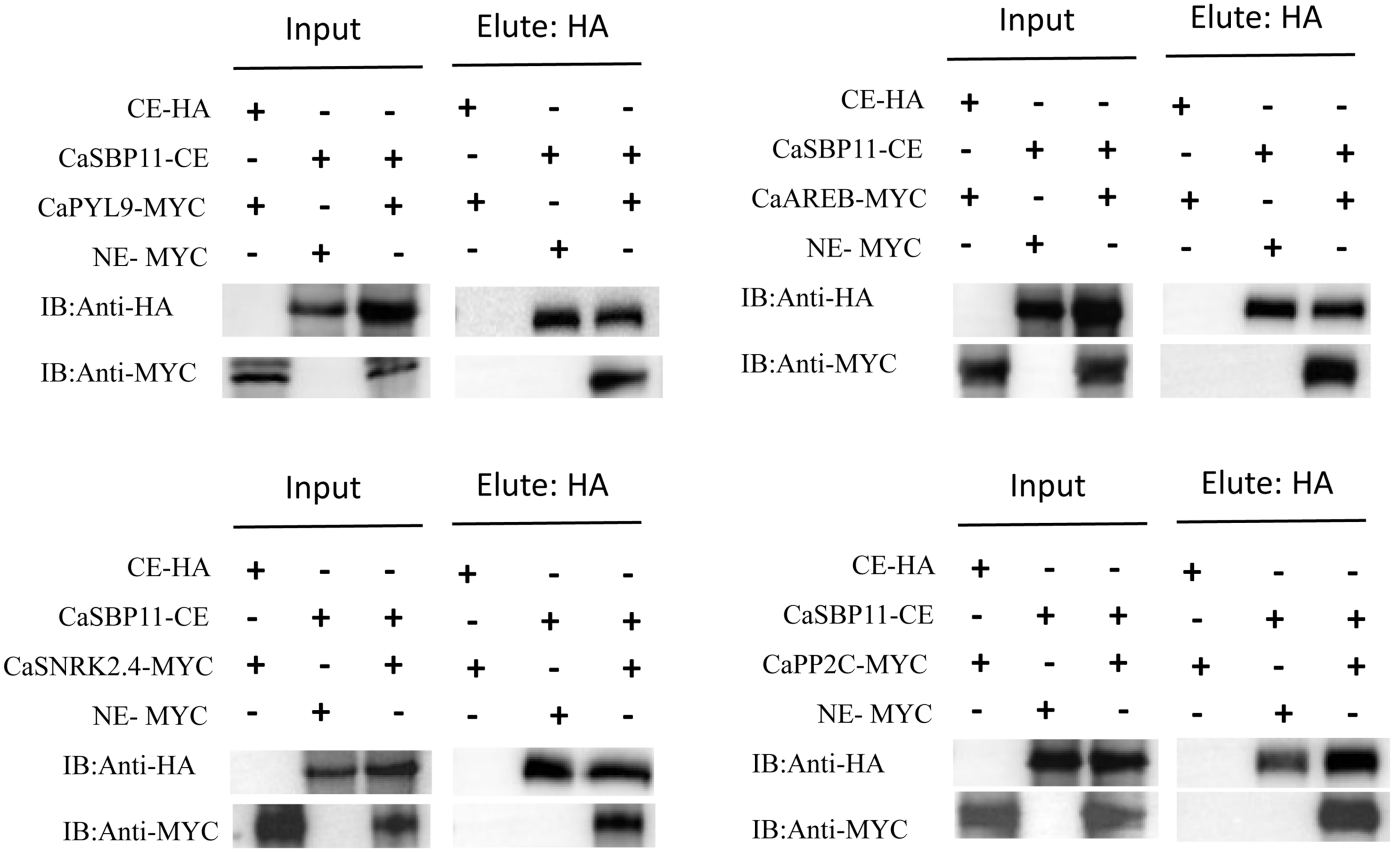
Co-immunoprecipitation (Co-IP) assays of CaSBP11 and CaPP2C, CaPYL9, CaSNRK2.4, and CaAREB. *N.benthamiana* leaves co-expressing CaPYL9-MYC and CaSBP11-CE, CaAREB-MYC and CaSBP11-CE, CaSnRK2.4-MYC and CaSBP11-CE, CaPP2C-MYC and CaSBP11-CE respectively were subjected to protein immunoprecipitation using the anti-MYC antibody.

In the previous study, it was found that under non-stress conditions, core ABA signaling cascade genes (*CaPP2C*, *CaPYL9*, *CaSNRK2.4*, *CaAREB*) exhibited lower expression levels in CaSBP11-silenced plants when compared to controls. Conversely, this trend was reversed for *CaSBP11* overexpressed lines (*NbPP2C*, *NbAREB*, *NbSNRK2.4*, *NbSRK2E*). Therefore, we speculate that CaSBP11 may regulate the plant’s response to drought stress through interactions with CaPP2C, CaPYL9, CaSNRK2.4, and CaAREB. Further research was conducted on this hypothesis. The BiFC experiments demonstrated that CaSBP11 interacts with CaPP2C, CaPYL9, CaSNRK2.4, and CaAREB in the nucleus. This was evidenced by a significant YFP signal in the nucleus, while no YFP signal was observed in the control samples (**Figure 7**, **Supplementary Figures 7**-**9**). We also performed Co-IP assays by co-expressing CaPYL9-MYC and CaSBP11-CE, CaAREB-MYC and CaSBP11-CE, CaSnRK2.4-MYC and CaSBP11-CE, CaPP2C-MYC and CaSBP11-CE in *N. benthamiana* leaves respectively (**Figure 8**). These results indicated that CaSBP11 interacts with CaPP2C, CaPYL9, CaSNRK2.4, and CaAREB. Additionally, CaSBP11 may participate in the response of pepper to drought stress by interacting with these genes.

## Discussion

The plant-specific SBP-box gene family comprises 15 members in pepper, including *CaSBP11*. Our previous research indicated that *CaSBP11* is located in the nucleus and contributes to the pepper’s defense against *Phytophthora capsici* infection via the salicylic acid (SA) signaling pathway (Zhang et al., 2020b). Nevertheless, its role in drought stress in peppers remains unclear.

Our previous study discovered that the transgenic *N. benthamiana* line overexpressing *CaSBP11* was sensitive to drought stress, prompting us to investigate *CaSBP11*’s function under drought conditions. Here, we found that the expression level of *CaSBP11* gene increases at 12 hours under drought stress (**Supplementary Figure 1**). To explore whether *CaSBP11* influences drought stress responses, we conducted gene silencing experiments. Reportedly, most SBP-Box genes are associated with plant morphogenesis and development. For example, the knockout of *SPL13* in *Solanum lycopersicum* increases lateral bud growth. Additionally, *SPL13* directly represses the transcription of *IPT1*, inhibiting bud growth in spl13 mutants (Chen et al., 2023). In maize, knockout of *Zmspl13* and *Zmspl29* delays the vegetative phase change and flowering time, while overexpression of *ZmSPL29* accelerates these processes, leading to early flowering (Yang et al., 2023a). *ARF3* binds to elements P1 and P2 of the SPL promoter, efficiently blocking AG-induced *SPL* activation and causing abnormal phenotypes in the mutants (Yang et al., 2023b). However, CaSBP11-silenced plants and control plants exhibited no discernible phenotype (**Supplementary Figure 2**). This lack of phenotype may be due to silencing not completely abolishing the gene’s function as a mutation would, or to *CaSBP11* not affecting pepper growth and development. Nonetheless, this necessitates further empirical validation. Silencing *CaSBP11*resulted in increased drought tolerance in plants (**Figure 1A**). Previous research has shown that silencing *CaSBP13* also enhances drought tolerance in pepper plants (Zhang et al., 2024). Both *CaSBP11* and *CaSBP13* belong to the SBP-box gene family in pepper (Zhang et al., 2016). Additionally, *CaSBP11* and *CaSBP13* negatively regulate pepper’s defense response to *Phytophthora capsici* infection (Zhang, 2020). Evolutionary analysis of SBP-box family genes in *Arabidopsis*, rice, and tomato revealed that *CaSBP11* and *CaSBP13* belong to different groups and are evolutionarily distant. Furthermore, an analysis of tandem duplications within the pepper SBP-box gene family showed that there are no interchromosomal segmental duplications between *CaSBP11* and *CaSBP13* (Zhang et al., 2016). Silencing either *CaSBP11* or *CaSBP13* genes improved the plant’s response to drought stress (Zhang et al., 2024). Hence, it is speculated that there is no functional redundancy between *CaSBP11* and *CaSBP13.* It has been demonstrated that drought induces excessive ROS production, leading to structural damage (Sewelam et al., 2016). ROS scavengers such as SOD, POD, and CAT efficiently convert surplus and detrimental ROS to harmless water under stress conditions (Noctor and Foyer, 1998; Xu et al., 2016).

In this investigation, the activity of CAT and POD was found to be significantly higherin CaSBP11-silenced plants compared to control plants. Conversely, the peak time for SOD activity was observed to be earlier in control plants than in CaSBP11-silenced plants (**Figure 1C**). Concurrently, the accumulation of H_2_O_2_ and O_2_^-^ in CaSBP11-silenced plants significantly declined compared to controls (**Figures 2A–F**). Additionally, the expression level of *CaAPX1*, *CaPOD*, *CaCAT2*, and *CaSOD*, which are linked to ROS-scavenging enzymes was detected post-drought stress after 4 days. Specifically, these genes were more highly expressed in *CaSBP11*-silenced plants compared to controls (**Figure 2G**). It has been reported that exposing soybeans to varying degrees of drought stress during the early flowering phase leads to an enhancement in H_2_O_2_ levels alongside a fluctuating pattern for SOD, POD, and CAT levels (Song et al., 2022). Similarly, inducing overexpression of *TaFDL2-1A* in wheat resulted in elevated SOD and GPX activity after drought stress, indicating a superior ability to counteract ROS in wheat plants (Wang et al., 2022). Furthermore, studies suggest that emphasized expression of *BpSPL9* can reinforce ROS scavenging by stimulating POD and SOD enzymes, thereby enhancing plant resistance to drought stress (Ning et al., 2017). O*sSPL10* overexpression is crucial for drought tolerance by managing ROS generation in rice (Li et al., 2023). Conversely, *TaSPL6-A* over-expression in wheat compromised drought resilience, presenting a significant surge in ROS (Zhao et al., 2024).

Furthermore, this research reveals that the number of stomata increases in both CaSBP11-silenced plants and controls, with notably higher numbers in the controls (**Figures 3A, B**). Additionally, the aperture of the stomata becomes smaller, but it is significantly higher in CaSBP11-silenced plants compared to the control plants (**Figures 3D, E**). Moreover, the ABA levels augmented in both CaSBP11-silenced and control plants, with significantly greater levels evident in CaSBP11-silenced plants (**Figure 3C**). Besides, it has been reported that ABA participates in plant responses to drought stress by inducing stomatal closure to minimize transpirational water loss and activating drought responsive genes, ultimately enhancing drought tolerance (Koh et al., 2023). During drought stress, ABA levels rise, causing a decrease in stomatal aperture and an increase in stomatal density (Jalakas et al., 2018; Geng et al., 2023; Hasanuzzaman et al., 2023; Lim et al., 2024). For instance, in CaJAZ1–03 gene-silenced plants, ABA can induce stomatal closure, resulting in a decrease in stomatal aperture. Moreover, *CaJAZ1–03* dampens abscisic acid (ABA) signaling and drought stress responses (Koh et al., 2023). *CaDeSI2* reduces the stability of the PP2C protein CaAITP1, a core component of ABA signaling, through deSUMOylation, thereby positively regulating drought stress tolerance and ABA-induced stomatal closure (Joo et al., 2024). Under drought stress, both the RcNAC091-silenced plants and the control plants exhibited an increase in stomatal density, and *RcNAC091* improves drought tolerance in an ABA-dependent manner. Furthermore, *RcNAC091* can bind to the promoter of *RcWRKY71*, regulating its function during drought stress. Similarly, both the RcWRKY71-silenced plants and the control plants showed an increase in stomatal density. *RcWRKY71* positively regulates the plant’s response to drought stress by modulating genes related to the ABA signaling pathway. Additionally, *RcWRKY71* might facilitate the ABA-dependent pathway during drought stress (Geng et al., 2023). Besides, CaMEKK17 can interact with PP2C, a core component of the ABA signaling pathway, and positively regulate plant tolerance to drought stress by impairing ABA-mediated stomatal closure (Lim et al., 2024). Furthermore, *OsSPL10* in rice negatively regulates drought stress responses by controlling stomatal movements (Li et al., 2023). Based on these results, we hypothesized that *CaSBP11*’s role in drought tolerance may be linked to the ABA signaling pathway. We subsequently examined these pivotal gene expressions, and discovered elevated expression of *CaPYL9*, *CaAREB*, *CaPP2C* and *CaSNRK2.4* in drought-stressed CaSBP11-silenced plants (**Figure 3F**). Of note was the significant reduction of these gene expressions in CaSBP11-silenced plants, which was uninfluenced by other factors, suggesting that the function of *CaSBP11* under drought stress may be related to the ABA signaling pathway (**Figure 3F**). Reportedly, when plants are challenged by adversity, ABA levels within plants rise. This ABA binds to its receptor *PYL*, initiating a reaction with *PP2C* that dephosphorylates *SnRK2*, thus resetting the inhibitory influence of *PP2C* on *SnRK2*. Phosphorylated *SnRK2* in turn modulates downstream transcription factors like *AREB*, triggering ABA signal transduction (Kim et al., 2022).

Besides, *CIPK1* interacts with and phosphorylates most ABA receptors at the evolutionary conserved site equivalent to PYL4 Ser129, thereby attenuating their activation and enhancing *PP2C* activity under normal conditions. During drought stress, ABA inhibits CIPK1-induced phosphorylation of *PYLs*, preventing them from fully responding to ABA signaling and enabling survival within challenging environmental conditions (You et al., 2023). Moreover, CaDeSI2 interacts with CaAITP1, a member of Group A PP2C proteins, enhancing drought resilience in pepper (Joo et al., 2024). The CaSnRK2.4 protein binds and phosphorylates the CaNAC035 protein, which enhancing cold tolerance via *CaSnRK2.4* dependence in pepper (Zhang et al., 2023). Additionally, MicroRNA miR156 is known to regulate SBP family genes in plants. Using the psRNATarget website (http://plantgrn.noble.org/psRNATarget/), we predicted that the *CaSBP11* gene contains miR156 target sequences. It has been reported that the interaction between miRNAs and transcription factors coordinates various signaling pathways in plants, including those mediated by ABA and non-ABA (Singroha et al., 2021). For instance, the potential involvement of miRNAs in ABA-dependent drought responses was evident when it was observed that the hyl1 mutant showed hypersensitivity to both ABA and drought (Lu and Fedoroff, 2000). The miRNA156 inhibits the transcription of miRNA172b through *SPL9* and, redundantly, through *SPL10*. Moreover, miRNA172 can reduce plants sensitivity to drought stress (Wu et al., 2009; Han et al., 2013; Singroha et al., 2021). Additionally, in *alfalfa*, *SPL3* is regulated by miR156. Moderate levels of miR156 transcripts are sufficient to enhance drought tolerance in *alfalfa* by silencing *SPL13* and increasing *WD40–1* expression. However, excessive overexpression of miR156 results in drought susceptibility. Furthermore, *SPL13* acts as a direct regulator of *DFR*, which is itself regulated by *WD40-1* (Arshad et al., 2017; Feyissa et al., 2019). Transgenic plants show enhanced tolerance to drought and salt stress through the expression of the miR156-SPLs-DFR network (Cui et al., 2014; Jeyakumar et al., 2020).

In order to validate *CaSBP11*’s involvement in plant drought stress response, we engineered *N. benthamiana* overexpressing *CaSBP11*. These *CaSBP11* overexpression lines exhibited increased drought sensitivity compared to their wild-type counterparts, with elevated ROS accumulation, MDA content, and relative electrical conductivity (**Figures 4**, **5**). Similarly, the expression levels of *NbSOD* and *NbCAT* in the *CaSBP11* overexpressors were significantly reduced after drought stress, corroborating findings from previous studies on pepper (**Figures 2G**, **5G**). In contrast, the expression of *NbPOD* and *NbAPX* increased; with the *CaSBP11* overexpression lines showing higher levels than wild-type plants (**Figure 5G**). This expression pattern is inconsistent with the results observed in pepper ((**Figures 2G**, **5G**)). It has been reported that under severe salt stress, the APX in the roots of *Leymus chinensis* increases significantly, while POD activity in the leaves increases, jointly eliminating ROS (Li et al., 2017). The expression of ROS-scavenging related genes is closely associated with the activity of ROS-scavenging enzymes. Thus, the contrasting expression patterns of *NbPOD* and *NbAPX* compared to those in pepper may be attributed to differences in crop species and the varying degrees of drought stress experienced by the plants. Moreover, under drought stress, both *CaSBP11* overexpression and wild-type plants showed an increase in stomatal density in their leaves compared to the control. However, the increase in stomatal density in wild-type plants was not significant (**Figure 6B**). In contrast, in pepper plants, the stomatal density of TRV2 plants under drought stress was significantly higher than that of the control TRV2 plants (**Figure 3B**). This difference may be attributed to the varying drought tolerance among different crop species. Furthermore, studies by Wang et al. (2010) have demonstrated that drought stress results in an increase in stomatal density and a decrease in stomatal aperture in the leaves of most plants, while stomatal distribution varies according to the species and the severity of the stress. Additionally, on day 4 of drought stress, the transcript levels of *NbPYL9*, *NbPP2C*, *NbAREB* and *NbSNRK2.4* in the *CaSBP11* overexpressing plants markedly declined compared to wild-type, corroborating pepper research predictions (**Figures 3F**, **6G**). Beyond this, under non-treatment conditions, the expressions of *NbAREB*, *NbPP2C*, *NbSNRK2.4*, and *NbSRK2E* in the *CaSBP11* overexpressors significantly exceeded those in the wild-type plants, echoing prior studies on pepper (**Figures 6G**, **3F**). Furthermore, ABA levels were elevated in both *CaSBP11* overexpressing and wild-type plants; however, the ABA content in the *CaSBP11* overexpressors was significantly lower than that in wild-type plants (**Figure 6C**). Notably, seed germination percentage and root length both decreased under fluctuating ABA conditions. Nevertheless, the wild-type exhibited a stronger ABA response than the plants overexpressing *CaSBP11* (**Supplementary Table 2**, **Supplementary Figure 5**). Moreover, we found that CaSBP11 interacts with CaPP2C, CaPYL9, CaSNRK2.4, and CaAREB in the nucleus (**Figures 7**, **8**, **Supplementary Figures 7**-**9**). Therefore, according to Fidler et al.’s findings regarding the ABA signaling pathway’s core components (*PYL*, *PP2C*, *SnRK2*, and *AREB*) and their roles therein (Fidler et al., 2022), combined with our research data, we speculated that under drought stress, the ABA content in the plant increases. Besides, ABA can inhibit the expression of the *CaSBP11* gene, thereby may reducing the interaction between CaSBP11 and CaPP2C, CaPYL9, CaSNRK2.4, and CaAREB. This inhibition promotes the expression of the *CaPYL9*, *CaSNRK2.4*, and *CaAREB* genes, initiating the ABA signaling pathway response and enhancing plant drought tolerance. However, further experimental verification is required.

## Conclusions

In summary, the expression level of *CaSBP11* gene is upregulated by drought stress at 12 hours post-treatment in pepper. Silencing *CaSBP11* enhances drought tolerance, correlating with reduced ROS content compared to control plants. Conversely, overexpressing *CaSBP11* in *N. benthamiana* increases the plant’s susceptibility to drought stress and the ROS accumulation compared to wild-types. Remarkably, drought-induced upregulations of *CaAPX1*, *CaCAT2*, *CaSOD*, and *CaPOD* transcripts in CaSBP11-silenced plants surpasses control levels. Conversely, post-stress, expression levels of *NbCAT1*and *NbSOD* are significantly reduced in *CaSBP11* overexpressors. Notably, under non-stress conditions, core ABA signaling genes (*CaPP2C*, *CaPYL9*, *CaSNRK2.4*, and *CaAREB*) exhibited lower expression in CaSBP11-silenced plants compared to the controls. Conversely, this trend was reversed in CaSBP11-overexpressing lines (*NbPP2C*, *NbAREB*, *NbSNRK2.4*, and *NbSRK2E*). Besides, CaSBP11 interacts with CaPP2C, CaPYL9, CaSNRK2.4, and CaAREB in the nucleus. These results suggest that*CaSBP11* plays a negatively role in plant drought tolerance, likely tied to ABA and ROS signaling. Nonetheless, additional research is imperative to elucidate these mechanisms.

## Data Availability

The original contributions presented in the study are included in the article/**Supplementary Material**. Further inquiries can be directed to the corresponding author.
