# Supplementary material for: The CaSBP11 gene functions as a negative regulator in pepper drought stress
Source: Front Plant Sci. 2025 Apr 28;16:1497425. doi: 10.3389/fpls.2025.1497425 (PMC12066515; doi:10.3389/fpls.2025.1497425)
Supplement: Supplementary file 1 [file DataSheet1.doc]

**SUPPLEMENTARY TABLES AND FIGURES**

**Supplementary Table 1.** Vectors' construct and quantitative PCR primer sets and their sequences.

| **Oligo Name** | **Primer Abbreviation** | **Primer Sequence (5^’-^3’)** |
| --- | --- | --- |
| ***CaSBP11*** | CaSBP11-2307-GFP-F | CGGGATCCATGGAGTCTTGGAGTTATTTCTCAGG |
|  | CaSBP11-2307-GFP-R | TCCCCCGGGGCAGTGATTCTAAGGCCGGG |
|  | CaSBP11-VIGS-F | CGGGATCCAGGACTGCCTGCCGTAACAAC |
|  | CaSBP11-VIGS-R | GGGGTACCGACGAGCCCTGTGATTGAGATG |
|  | RTCaSBP11-VIGS-F | CATCTCAATCACAGGGCTCG |
|  | RTCaSBP11-VIGS-R | CATTACTATCCTGCTTCACTTGC |
|  | CaSBP11-CE-F | GGCCTGGCGCGCCACTAGTGGATCCATGGAGTCTTGGAGTTATTTCTCAGG |
|  | CaSBP11-CE-R | ATGGGTACATCCCGGGAGCGGTACCAGTGATTCTAAGGCCGGGAAAAGC |
| ***CaUBI3*** | CaUBI3-F | TGTCCATCTGCTCTCTGTTG |
|  | CaUBI3-R | CACCCCAAGCACAATAAGAC |
| ***CaAPX1*** | CaAPX1-F | AGAGGACAAGCCAGAACCAC |
|  | CaAPX1-R | CCTTGTCTGATGGCAACTGT |
| ***CaCAT2*** | CaCAT2-F | GAAGCCAAATCCTAAGTCCC |
|  | CaCAT2-R | CCAACTCGGATTGCCTCTT |
| ***CaSOD*** | CaSOD-F | TATGGAGCCTTAGAACCTGC |
|  | CaSOD-R | CCATTGAACTTGATAGCACCT |
| ***CaPOD*** | CaPOD-F | TCCTCCTCCTACTTCTAACC |
|  | CaPOD-R | ACAGACCTCTTTTGCTCACT |
| ***CaPYL9*** | CaPYL9-F | CGTTGAAAGGAGGAGT |
|  | CaPYL9-R | ACAAGATGAACAGGAGC |
|  | CaPYL9-NE -F | GGAGAGAACACGGGGGACTCTAGAATGGTCAGCATAATGAACAACGTTGAAAG |
|  | CaPYL9-NE -R | CATCCCGGGAGCGGTACCCTCGAGCATTCTGTCGATAGGCTCCATATGG |
| ***CaPP2C*** | CaPP2C-F | GTGATTCTCGTGCTGTGC |
|  | CaPP2C-R | CAGGCAGTCTCGTTTGAT |
|  | CaPP2C-NE -F | GGAGAGAACACGGGGGACTCTAGAATGGCTGGCATGTGTTGTGGTG |
|  | CaPP2C-NE -R | CATCCCGGGAGCGGTACCCTCGAGTAGCTTTCTCAAATCAACCACGACG |
| ***CaAREB*** | CaAREB-F | GGAAATGAGCCACCAG |
|  | CaAREB-R | CCCACCAATCCCAGTA |
|  | CaAREB-NE -F | GGAGAGAACACGGGGGACTCTAGAATGGGGAGTAATTATAATTTCAAGAACTTTGG |
|  | CaAREB-NE-R | CATCCCGGGAGCGGTACCCTCGAGCCACGGACCAGTCTGTGTC |
| ***CaSNRK2.4*** | CaSNRK2.4-F | TGAGAATGTAGCAAGGGAG |
|  | CaSNRK2.4-R | GCAACAGGGACGACTT |
|  | CaSNRK2.4-NE -F | GGAGAGAACACGGGGGACTCTAGAATGGAAAGATACGAGCTTGTGAAGG |
|  | CaSNRK2.4-NE -R | CATCCCGGGAGCGGTACCCTCGAGGCTGAGACGAACATCCCCTAAG |
| ***Nbactin-97*** | Nbactin-F | TATGGAAACATTGTGCTCAGTGG |
|  | Nbactin-R | CCAGATTCGTCATACTCTGCC |
| ***NbAPX*** | NbAPX-F | CCAAGGGTTCTGACCATCTG |
|  | NbAPX-R | GCATAGTCGGCAAAGAAAGC |
| ***NbCAT1*** | NbCAT1-RT-F | TCTATTGTGGTTCCAGGGGTTT |
|  | NbCAT-RT-R | CACCCACCGACGAATAAAGC |
| ***NbSOD*** | NbSOD-RT-F | GCAGACGGACCTTAGCAACA |
|  | NbSOD-RT-R | TGGCGACGGTAGGAGCAT |
| ***NbPOD*** | NbPOD-RT-F | AGGCTCAGGGGACAACAACT |
|  | NbPOD-RT-R | TCACAAAATCAGTGGCGAAA |
| ***NbPYL9*** | NbPYL9-F | ACCGTTTGTTAGCAGG |
|  | NbPYL9-R | CGTCTTTAGTATTCCCATCT |
| ***NbPP2C*** | NbPP2C-F | TGTCGGAATCTGATGTTT |
|  | NbPP2C-R | ACGGGTGAACTGCTACT |
| ***NbAREB*** | NbAREB-F | TTTCGTTCAAGGTGCTG |
|  | NbAREB-R | CATTCTCCTTTGCCTCC |
| ***NbSNRK2.4*** | NbSNRK2.4-F | TTCCCGTTCAGTCTCAGGCTT |
|  | NbSNRK2.4-R | TGTGCCTGCTTCACTTGCTTT |
| ***NbSRK2E*** | NbSRK2E-F | TCTAAGTCCTCGGTGTTG |
|  | NbSRK2E-R | TTCGCTGTATTGTCTTGC |

**
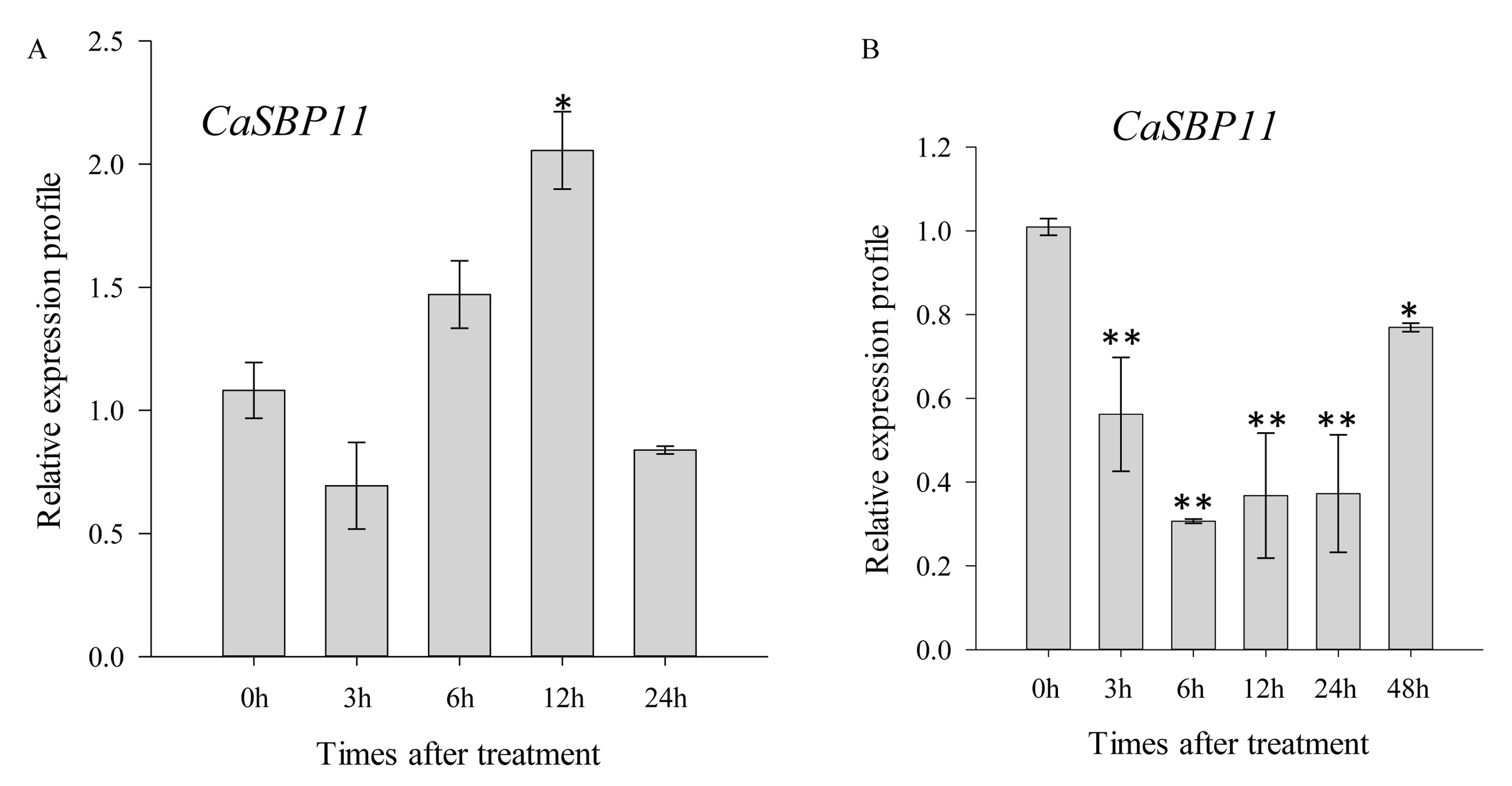
**

**Supplementary Figure 1.** Expression profiling of *CaSBP11* gene under drought and ABA stress in pepper. (A) Following 20% Polyethylene glycol (PEG6000) treatment, *CaSBP11* expression was assessed at 0, 3, 6, 12, 24 hours post-treatment. (B) Following 20μM ABA treatment, *CaSBP11* expression was evaluated at 0, 3, 6, 12, 24, 48 hours post-treatment. * and ** denote significant differences at *P* ≤ 0.05 and *P* ≤0.01 respectively. Mean values and SDs for three replicates are displayed.

**
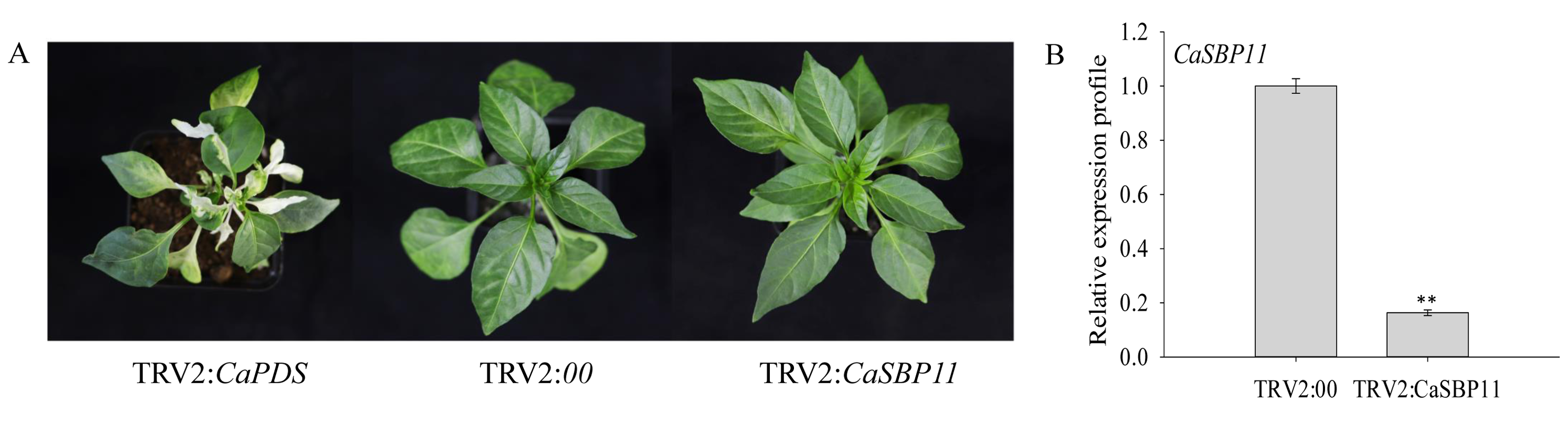
**

**Supplementary Figure 2.** Phenotype and silencing efficacy of CaSBP11-silenced plant. (A) Plant phenotype following *CaSBP11* silencing. Images obtained forty days post-injection, pot diameter 7 cm. (B) *CaSBP11* silencing efficacy in silenced versus negative control plants. ** denotes significant disparity at *P* ≤ 0.01. Mean values and SDs for three replicates are displayed.

**
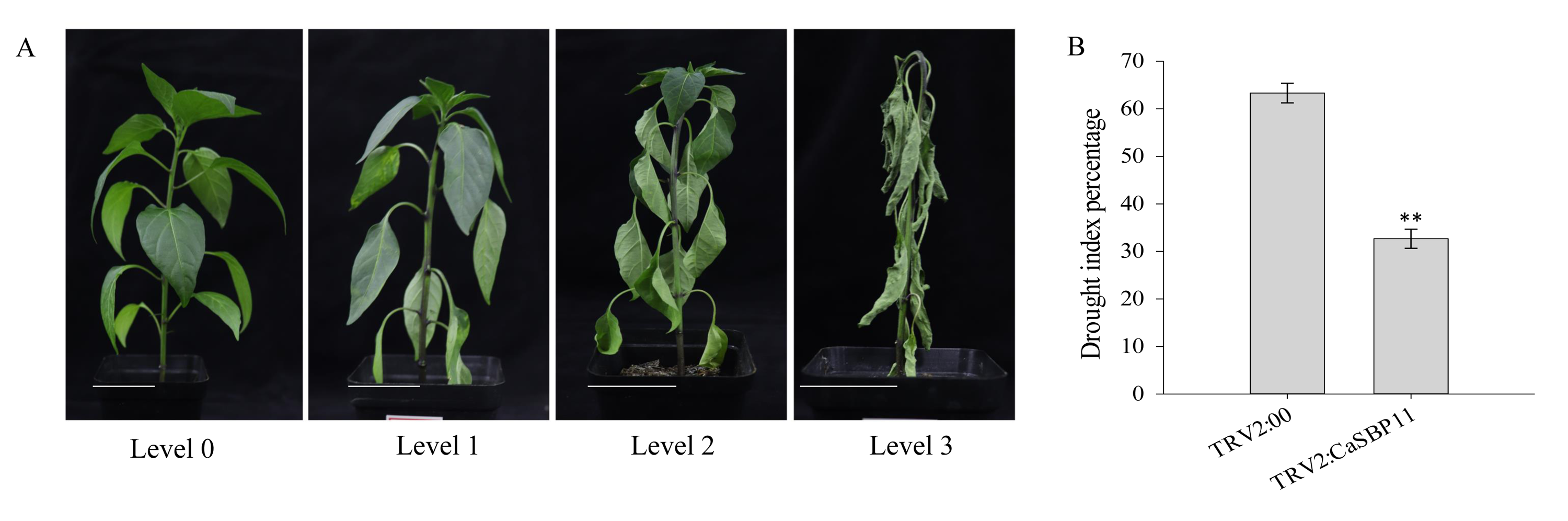
**

**Supplementary Figure 3.** Statistical analysis of percent drought indices in CaSBP11- silenced and control plants after drought stress 5 days. (A) Classification standards for drought phenotypes in CaSBP11-silenced and control plants. Level 0, no symptoms; level 1, lower leaves of plant wilted; level 2, all plant leaves except the growing point wilted; level 3, entire plant wilted. Scale bar, 3.5cm. (B) Drought index percentage analysis of CaSBP11-silenced and control plants. ** denotes significance at *P* ≤ 0.01. Mean values and SDs for three replicates are displayed.


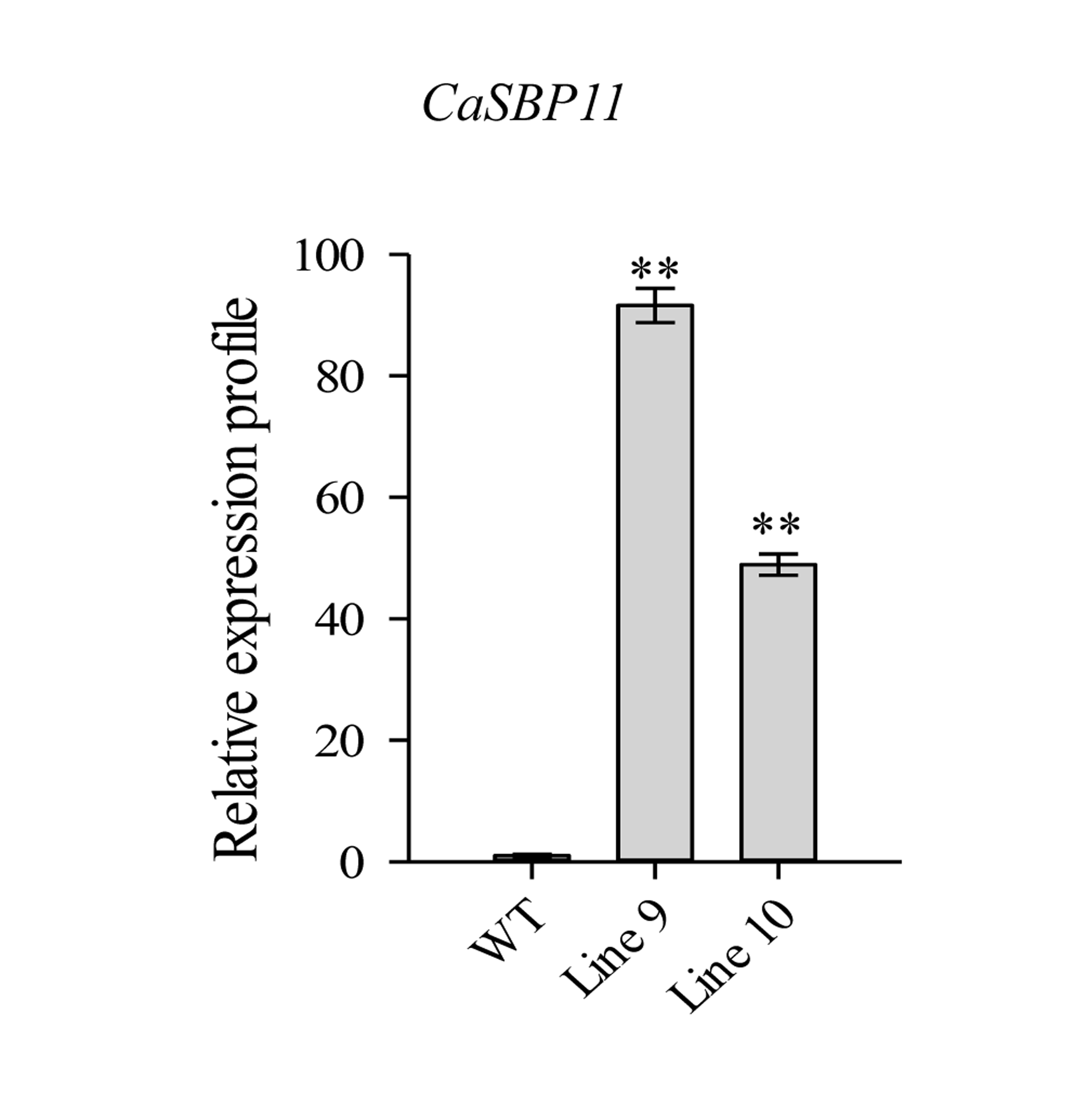


**Supplementary Figure 4.** *CaSBP11* gene expression in overexpressing *CaSBP11* and wild-type (WT) lines of *Nicotiana benthamiana*. ** denotes significance at *P* ≤ 0.01. Mean values and SDs for three replicates are displayed.

**Supplementary Table 2.** Statistic on germination rate for *CaSBP11* overexpressed plants under ABA treatment.

| Time | Plants | | 0g/LABA | | --- | | 0.1g/LABA | 0.5g/LABA | 1g/LABA |
| --- | --- | --- | --- | --- | --- |
| 3d | WT | 0.988±0.016a | 0.523±0.043c | 0.111±0.059e | 0.000±0.000f |
|  | Line 9 | 0.933±0.019a | 0.711±0.011b | 0.233±0.018d | 0.023±0.003f |
|  | Line 10 | 0.957±0.017a | 0.733±0.033b | 0.289±0.016d | 0.000±0.000f |
| 5d | WT | 1.000±0.000a | 1.000±0.000a | 0.822±0.027b | 0.586±0.028d |
|  | Line 9 | 1.000±0.000a | 1.000±0.000a | 0.933±0.017a | 0.711±0.029c |
|  | Line 10 | 1.000±0.000a | 1.000±0.000a | 0.956±0.018a | 0.767±0.041c |
| 10d | WT | 1.000±0.000a | 1.000±0.000a | 1.000±0.000a | 1.000±0.000a |
|  | Line 9 | 1.000±0.000a | 1.000±0.000a | 1.000±0.000a | 1.000±0.000a |
|  | Line 10 | 1.000±0.000a | 1.000±0.000a | 1.000±0.000a | 1.000±0.000a |

Letters indicate significant differences at *P* ≤ 0.05.


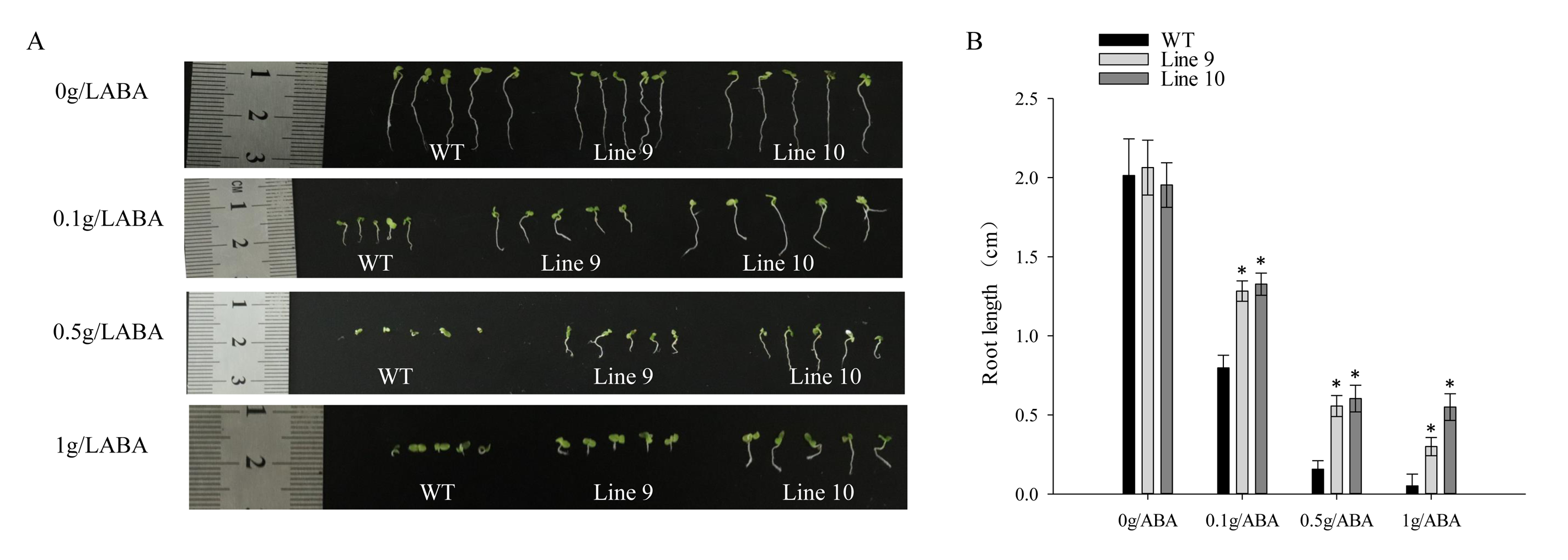


**Supplementary Figure 5.** The root length of *CaSBP11* overexpressed and wild-type plants under various ABA applications. (A) Morphological evaluation of the plants following 10 days of distinct ABA treatments. (B) Root length of the plants, post-10 days of treatment with variable ABA concentrations. * denotes significance at *P* ≤ 0.05. Mean values and SDs for three replicates are displayed.


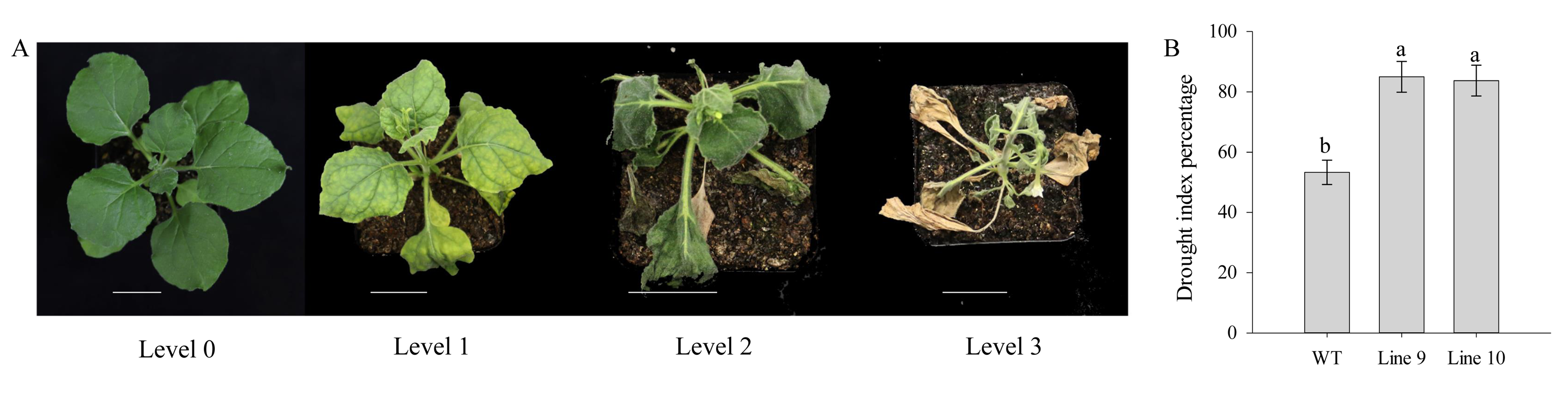


**Supplementary Figure 6.** Statistical analysis of percent drought indices in *CaSBP11* overexpression and wild-type plants after drought stress 13 days. (A) Classification standards for drought phenotypes in *CaSBP11* overexpression and wild-type plants after drought stress 13 days. level 0, no symptoms; level 1, wilting or yellowing of lower leaves; level 2, sublethal leaf death; level 3, plant death excluding the growing point .Scale bar, 3.5cm. (B) Drought index percentage analysis of *CaSBP11* overexpression and wild-type plants. Letters indicate significant differences at *P* ≤ 0.05. Mean values and SDs for three replicates are displayed.

**
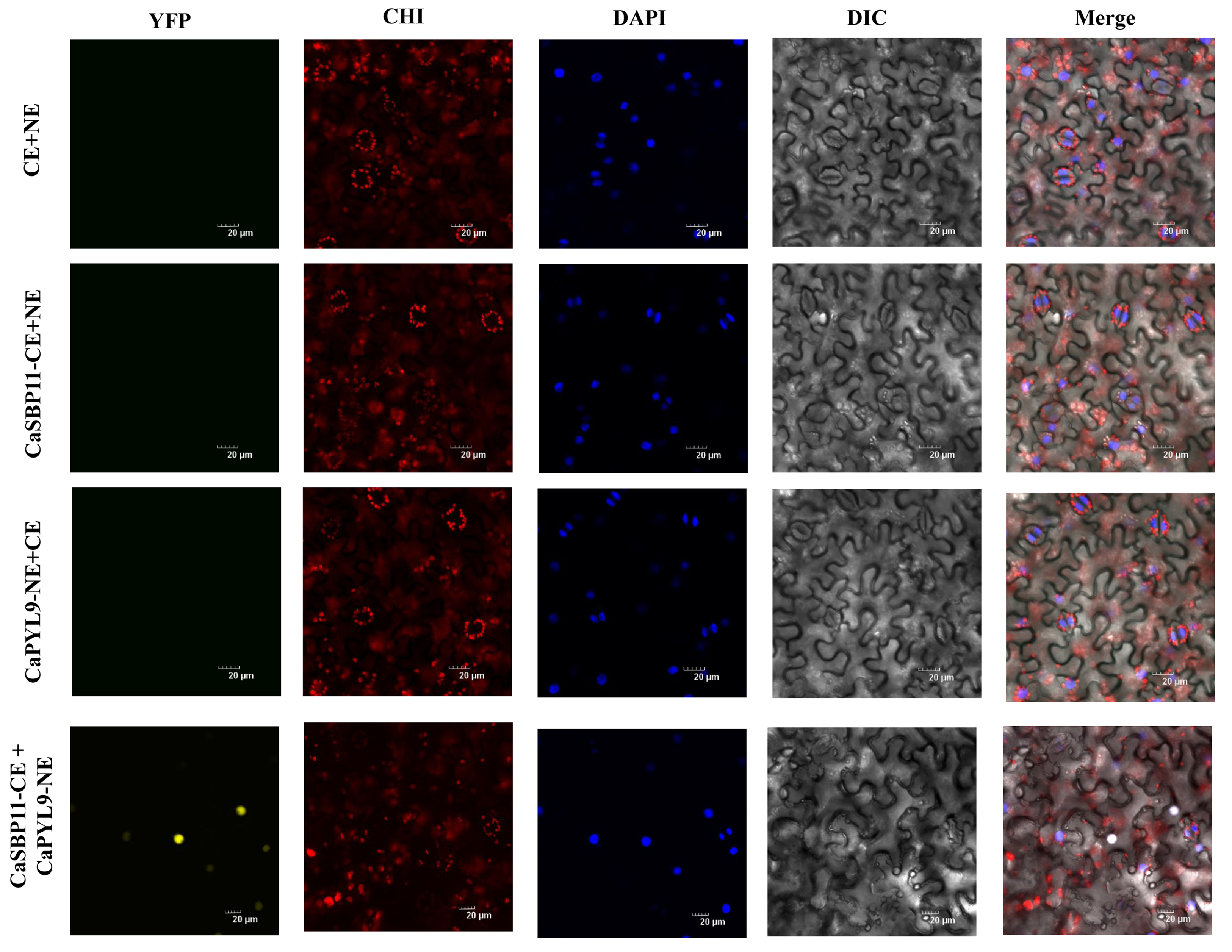
**

**Supplementary Figure 7.** BiFC Assay of CaSBP11 and CaPYL9. YFP represents the yellow fluorescent field, CHI represents the chloroplast autofluorescence field, DAPI represents the DAPI field (nuclear staining), DIC represents the bright field, and Merge represents the overlay field. Excitation wavelengths: YFP field (515 nm), CHI field (488 nm), DAPI field (358 nm). Bar = 20 µM.


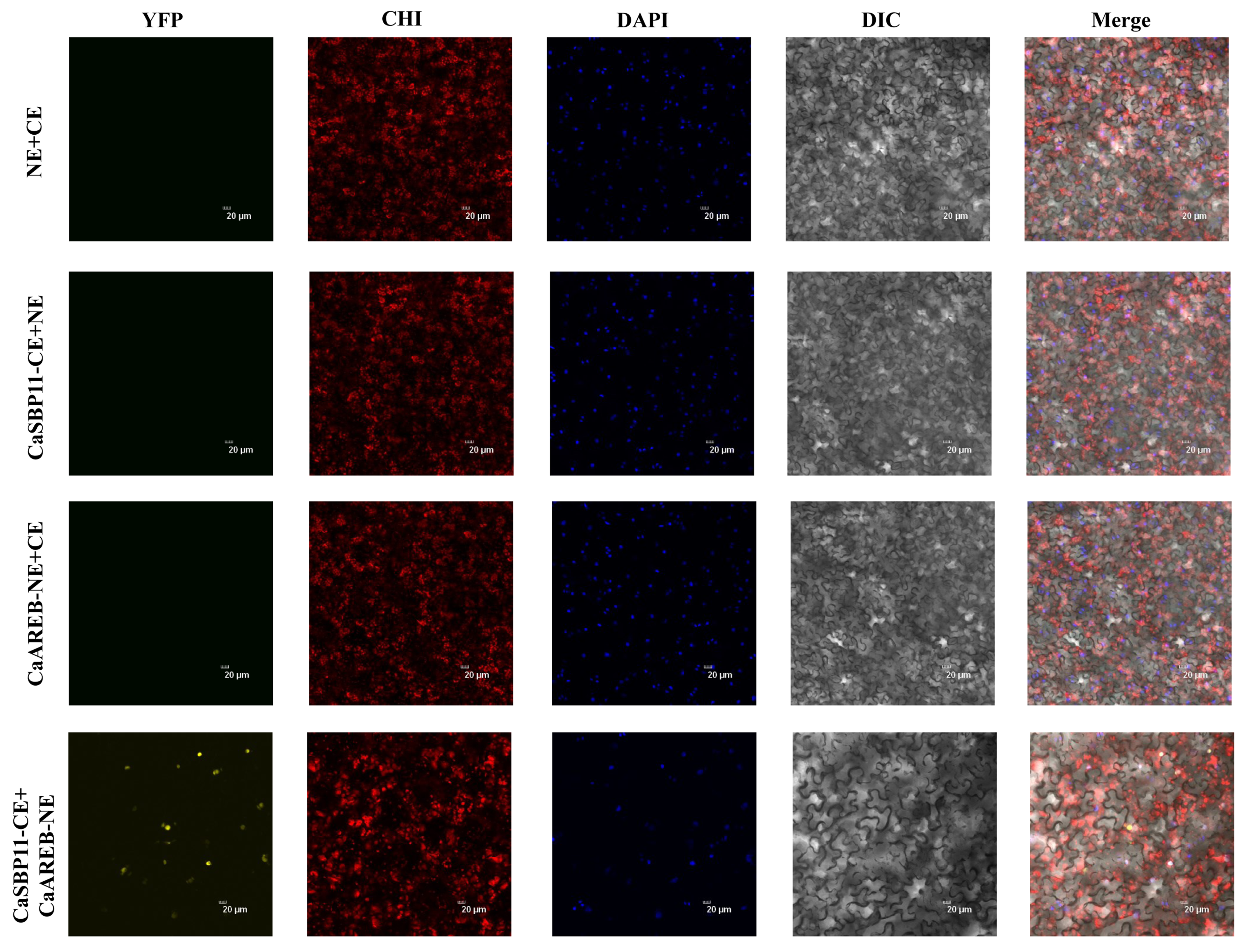


**Supplementary Figure 8.** BiFC assay of CaSBP11 and CaAREB. YFP represents the yellow fluorescent field, CHI represents the chloroplast autofluorescence field, DAPI represents the DAPI field (nuclear staining), DIC represents the bright field, and Merge represents the overlay field. Excitation wavelengths: YFP field (515 nm), CHI field (488 nm), DAPI field (358 nm). Bar = 20 µM.


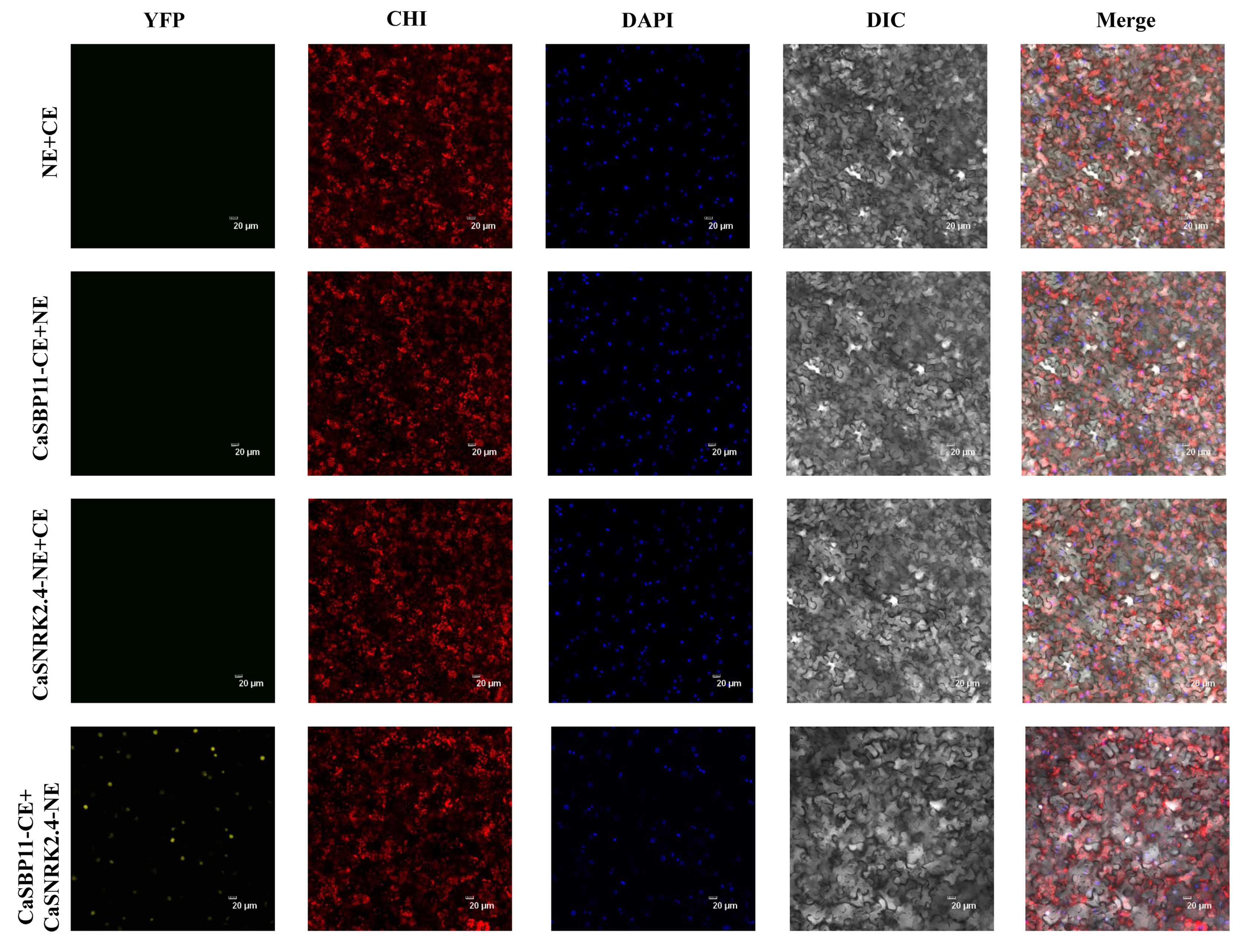


**Supplementary Figure 9.** BiFC Assay of CaSBP11 and CaSNRK2.4. YFP represents the yellow fluorescent field, CHI represents the chloroplast autofluorescence field, DAPI represents the DAPI field (nuclear staining), DIC represents the bright field, and Merge represents the overlay field. Excitation wavelengths: YFP field (515 nm), CHI field (488 nm), DAPI field (358 nm). Bar = 20 µM.
